# Regime-Dependent Elastic Displacement in Bio-Inspired Parametric Kirigami Structures: An Experimental Study of Geometric Parameter Effects

**DOI:** 10.3390/biomimetics11060427

**Published:** 2026-06-15

**Authors:** Tarek H. Mokhtar, Somaih M. Bakr, Qusai R. Khashman

**Affiliations:** DaVinciAT Robotics in Architecture Research Center, College of Engineering and Advanced Computing, Alfaisal University, Riyadh 11533, Saudi Arabia; sbakr@alfaisal.edu (S.M.B.); qkhashman@alfaisal.edu (Q.R.K.)

**Keywords:** Parametric Kirigami, elastic displacement, bio-inspired geometry, adaptive thin-sheet structures, compliant mechanisms, regime-dependent deformation, morphological adaptation

## Abstract

Biological thin-sheet systems, including leaves, insect wings, and flowering organs, achieve adaptive deformation through distributed compliance, segmentation, curvature, and controlled opening. Kirigami offers a bio-inspired route for translating such deformation logics into programmable thin-sheet surfaces; however, the geometric parameters that most strongly influence elastic displacement remain insufficiently quantified, especially across different loading regimes. This study investigates Bio-Inspired Regime-Dependent Parameter Selection in Parametric Kirigami through twenty-five laser-cut specimens spanning five boundary shapes and three thermoplastic substrates. Specimens were tested under two contrasting regimes: quasi-static tensile loading and gravity-drape loading. Elastic displacement was measured under eight-point boundary fixation and analyzed using regime-separated Pearson correlations, Bonferroni-corrected significance testing (α/18 = 0.0028), and shape-controlled partial correlations. Under tensile loading, the Number of Offsets (r = 0.807), Segments per Offset (r = −0.603), and outer-boundary void perimeter (r = 0.621) showed the strongest Bonferroni-robust associations with displacement. Under gravity-drape loading, effects were weaker and more curvature-sensitive, indicating that parameter relevance is not universal but regime-dependent. Within the tested parametric design space, the study provides an experimentally grounded basis for selecting Kirigami geometric parameters in thin-sheet structures whose adaptive deformation logic is analogous to compliant systems found in nature.

## 1. Introduction

Biological thin-sheet systems rarely deform by material softness alone. Leaves bend through venation and distributed compliance; insect wings deploy through patterned folding and segmented stiffness; flowering organs open through curvature, anisotropy, and localized flexibility. Kirigami offers a synthetic analog to these natural deformation logics: a thin sheet whose performance is programmed not by adding mechanisms, but by cutting geometry into the material itself. The design question is therefore not simply whether Kirigami can deform, but how its elastic displacement can be selected before fabrication. In bio-inspired adaptive surfaces, displacement is not a secondary consequence of form; it is the intended performance. What is needed is a parameter-level understanding of which geometric variables structure displacement, how strongly they do so, and whether their effects remain stable when loading conditions change.

This problem sits between two established lines of work. Research on cellular solids and lattice architectures shows that mechanical response depends strongly on structural organization, topology, connectivity, and relative density, rather than on constituent material alone [1,2,3]. Research on Kirigami shows that patterned cuts can enable large reversible deformation in thin sheets through hinge-like rotations and instability-driven buckling [4,5,6,7]. From a biomimetic lens, these studies indicate that deformation in cut-sheet systems is not a passive response, but an organized spatial effect produced by the distribution of openings, ligaments, boundaries, and compliant pathways.

Yet this body of knowledge remains incomplete for bio-inspired pre-fabrication parameter selection. Prior studies show that cut geometry influences stretchability, deformation magnitude, and post-buckling morphology [4,6,8,9], but they do not provide a systematic basis for distinguishing which geometric parameters are most relevant to elastic displacement within a shared parametric space. The literature is rich in reported outcomes—strain, stiffness, morphology, and deformation mode—but comparatively limited in guidance on how geometric variables might be prioritized, coordinated, or constrained before fabrication to achieve a target displacement.

This situates the present study relative to, rather than in competition with, predictive and analytical models of Kirigami mechanics. Closed-form and computational treatments of cut-sheet deformation seek to predict mechanical response from constitutive and geometric first principles; the present work instead adopts a statistical-association, screening-level stance, asking which geometric parameters covary most strongly and most stably with elastic displacement within a bounded design space and a specified loading regime. The two orientations are complementary rather than alternative: the contribution offered here is design-stage guidance for pre-fabrication parameter selection, not a competing mechanical model of how cut geometry generates deformation.

The difficulty becomes sharper when Kirigami is considered under more than one loading condition. Natural compliant systems do not deform independently of context: a leaf under self-weight, an insect wing during deployment, and a petal during opening each activate different combinations of compliance, curvature, and boundary constraint. Much of the existing Kirigami literature examines deformation under uniaxial tension or closely related loading contexts [4,6,10,11]. At the same time, work on mechanical metamaterials shows that structural response depends on architecture and boundary conditions, and that boundary programmability can emerge from architectural length scales [12,13]. Within Kirigami itself, Rafsanjani and Bertoldi (2017) [6] show that loading direction affects the morphology of buckling-induced three-dimensional patterns. These studies do not establish a general taxonomy of parameter roles across regimes, but they do indicate that geometric effects should not be assumed to remain invariant across loading and constraint conditions.

Accordingly, elastic displacement is treated here not as a fixed property of geometry alone, but as a regime-dependent performance outcome realized through the interaction of geometric organization and loading condition. From this standpoint, the design problem is not only to describe deformation after fabrication, but to select geometric parameters in advance relative to the intended condition of activation.

This paper addresses that problem through Bio-Inspired Regime-Dependent Parameter Selection in Parametric Kirigami. The selection procedure proceeds from the proposition that geometric parameters do not contribute equally to elastic displacement within a bounded design space. Instead, their relevance may differ, and may shift with the loading regime. The contribution of the study lies not only in identifying empirical parameter–displacement relationships, but in organizing those relationships into a form that supports design-stage reasoning for bio-inspired adaptive surfaces, compliant mechanisms, and deployable thin-sheet structures.

To investigate this, the study compares two contrasting regimes: quasi-static tensile loading, which engages internal hinge-like mechanisms through concentrated force paths, and gravity-driven loading, which distributes deformation through self-weight, boundary curvature, and bending. An exploratory experimental study was conducted using twenty-five parametrically generated Kirigami specimens spanning multiple shape categories and material substrates. Elastic response was measured as vertical displacement and analyzed through regime-separated correlation screening, Bonferroni-controlled significance testing (α/18 = 0.0028), and shape-controlled partial correlations. Within the present study, parameter influence is reported as the statistically robust covariation of a geometric parameter with elastic displacement within the tested design space and specified loading regime. The study contributes to the advancement of biomimetics by translating the regime-dependent compliance principles observed in natural thin-sheet systems (leaves, insect wings, flowering organs) into an experimentally quantified parameter hierarchy for an engineered Kirigami analog.

The study’s design contribution proceeds across three coordinated claims. First, it proposes a regime-separated empirical classification of geometric parameters according to their demonstrated capacity to structure elastic displacement within the tested parametric Kirigami design space, distinguishing Governing, Modulating, and Non-operative parameters under quasi-static tensile and gravity-driven loading, with three parameter–displacement associations surviving Bonferroni correction. Second, it translates these associations into Parameter-Specific Design Implications: bounded, actionable propositions specifying the loading regime under which the relationship holds, the direction of parameter adjustment, and the evidential robustness warranting its use. Third, it integrates these Parameter-Specific Design Implications into Bio-Inspired Regime-Dependent Parameter Selection in Parametric Kirigami, a sequential decision structure that links performance intent, regime activation, parameter hierarchy, dependency resolution, candidate generation, and configuration screening into pre-fabrication design logic. The procedure does not propose universal rules or exact displacement prediction. Rather, it supports elastic displacement as a selectable design variable within the tested design space, converting regime-dependent empirical association into actionable bio-inspired guidance for parametric Kirigami configuration.

## 2. Background and Theoretical Framing

### 2.1. Kirigami as a Geometry-Influenced Mechanical System

Research on Kirigami spans computational geometry, kinematics, and mechanics. Foundational contributions were primarily concerned with how planar sheets could be transformed into three-dimensional configurations through folding logics, tessellation strategies, and geometric patterning [14,15,16,17,18,19]. Related work examined the emergence of bistability and hidden degrees of freedom in folded structures [20]. In this body of work, geometry was treated mainly as a formal and kinematic medium for transformation rather than as a basis for evaluating displacement performance.

Subsequent mechanics-based studies shifted attention from transformation to response. Shyu et al. show that Kirigami-inspired patterned defects can substantially increase tensile stretchability in relatively rigid sheets [4]. Rafsanjani and Bertoldi (2017) show that mutually orthogonal cuts induce out-of-plane buckling under uniaxial tension, producing three-dimensional morphologies whose form depends on ligament instability and loading direction [6]. Related work on mechanical metamaterials shows more broadly that structural architecture can govern nonlinear response through buckling and related mechanism-like behavior [8,12,13,21,22].

Taken together, these studies indicate that deformation in Kirigami is strongly influenced by geometric configuration. However, they do not systematically differentiate the relative contribution of individual geometric variables to elastic displacement within a shared parametric design space.

### 2.2. Geometry, Topology, and Parameter Differentiation

The dependence of mechanical performance on structural organization is well established in cellular solids and architected materials. In cellular systems, topology, connectivity, and relative density influence whether deformation is bending- or stretching-dominated, thereby shaping stiffness-to-weight and strength-to-weight relationships [1,2,23]. In architected and metamaterial systems, behavior arises from the coordinated action of structural elements and their geometry-dependent modes of deformation [3,8,24].

This literature supports the view that geometric variables need not contribute uniformly to mechanical response. In porous and architected systems, response can vary with geometry and boundary conditions, and structural length scales can affect how deformation is realized near boundaries [12,13]. What follows from this work is not a predefined taxonomy of parameter roles, but a strong basis for expecting unequal relevance among geometric descriptors.

In parametric Kirigami systems, this unevenness becomes a central problem. Parametric models can expose numerous adjustable descriptors—offset count, offset spacing, segmentation density, boundary geometry, curvature, and related measures—yet their availability does not imply equal relevance to displacement. The issue is therefore not merely the number of variables, but their differentiation: which parameters act as primary contributors to displacement, which constrain or modulate that behavior, and which remain weak or inactive within a bounded design space and specified outcome. This differentiation remains largely unresolved in the literature on elastic displacement.

### 2.3. Conditional Realization of Displacement Under Loading Regime

A dominant strand of Kirigami mechanics has focused on uniaxial tension, where deformation mechanisms can be observed, modeled, and quantified with relative clarity [4,6]. Related work on Kirigami metamaterials also shows that engineered cuts can alter deformation characteristics and directional mechanical behavior [10].

At the same time, prior work indicates that realized deformation depends on loading and boundary conditions. Rafsanjani and Bertoldi show that loading direction affects the morphology of buckling-induced three-dimensional patterns [6]. Coulais et al. show that in mechanical metamaterials, boundary programmability and size effects arise from a characteristic architectural length scale [13]. Overvelde et al. show that compaction behavior in periodic porous structures depends on pore shape [12]. Read together, these studies support the interpretation that structural response is shaped by both geometry and the conditions under which that geometry is loaded and constrained.

For the present study, this means that parameter relevance should not be assumed to transfer automatically from one loading regime to another. A configuration that is highly responsive under tensile activation need not exhibit the same displacement behavior under gravity-driven deformation. Loading regime is therefore treated here not merely as a testing condition, but as part of the problem through which displacement is realized and interpreted.

### 2.4. Theoretical Framing Toward Governance

In this study, governance denotes an empirical ranking tier: the relative strength, consistency, and robustness with which a geometric parameter is statistically associated with elastic displacement within a specified loading regime and bounded design space. It is a ranking heuristic for design screening, not a claim about a physical governing mechanism intrinsic to the material system. Parameters are classified according to differentiated roles—Governing, Modulating, or Non-operative—based on the strength, consistency, and robustness of their observed associations. These classifications support the derivation of design guidelines and the construction of a procedure for adaptive geometric configuration.

### 2.5. Biomimetic Relevance of Kirigami Deformation Systems

Biological thin-sheet systems realize large, reversible deformation, deployability, and morphological adaptation through a limited repertoire of geometric mechanisms: distributed flexibility, hierarchical segmentation, anisotropic structural organization, curvature modulation, and localized compliance. The present study is not a direct biological replication model but a geometry-based abstraction of these adaptive deformation principles observed in natural compliant systems. This subsection situates the parametric Kirigami system examined in this paper within that biomimetic landscape, identifying the natural analogs whose deformation logics most closely correspond to the geometric parameters tested here. The correspondence is therefore strictly geometric: the present study does not model, and does not claim to reproduce, the heterogeneous, viscoelastic, or otherwise constitutive behavior of biological tissue, and all biomimetic statements in this paper are intended as functional–geometric analogies rather than claims of biological equivalence.

The first and most direct analog is leaf venation and differential flexibility in foliar tissues. In palm fronds, unfolding leaves, fern crosiers, and carnivorous plant lobes, large-scale deformation is mediated by hierarchically organized vein networks that produce locally variable compliance across the lamina: stiff along principal veins, compliant in the interstitial tissue, and curvature-tunable through preferential bending lines [25,26,27]. The result is a thin-sheet system that deforms not as an isotropic membrane but as a topologically organized field of compliance pathways. The geometric correspondence to the Kirigami configurations tested here is direct: the number of offset curves and their segmentation density define the multiplicity and continuity of compliance pathways within each specimen, while the boundary cut perimeter modulates edge flexibility in a manner mechanically analogous to marginal venation in leaves.

A second, closely related analog is insect wing folding. Beetle elytra and underlying hindwings, and the highly folded membranous wings of earwigs (Dermaptera), achieve compact storage and rapid deployment of thin, stiffness-variable sheets through engineered crease topologies and pre-programmed fold lines [28,29,30]. These systems are geometry-governed rather than material-governed: their deployability, bending stiffness, and reversibility emerge from the topology of the cut-and-fold pattern relative to the membrane thickness and boundary attachment. This is the family of biological systems most mechanically proximate to engineered Kirigami sheets: segmented, deployable, thin, and dependent on cut-and-crease geometry for their response. The parametric variables manipulated in the present study—offset architecture, segmentation, solid-bridge distribution, and boundary topology—are functionally comparable geometric abstractions of the parameters that control deployment and stiffness in folded insect wings. Beyond deployment, these elytral structures also integrate structure and function: the coupling geometry of beetle elytra unites mechanical deployability with a water-shedding, water-proofing role that shields the folded hindwing beneath, so that a single cut-and-fold architecture simultaneously governs deformation and surface-level fluid management [31]. It is this integration of geometric organization with functional performance, rather than form in isolation, that the present parametric abstraction seeks to operationalize.

A third analog is flower blooming and reversible petal motion. Petal opening and closing in many angiosperms is driven by differential growth, turgor change, or anisotropic tissue stiffness acting along curvature gradients [32,33,34]. Mechanically, this is a curvature-activated, regime-dependent deformation: the same petal geometry expresses different morphologies under self-weight loading, hydration change, or external mechanical perturbation. This is the closest biological analog to the gravity-drape regime examined here, where outer curvature emerges as a stronger geometric driver than internal cut architecture and where deformation is distributed across the specimen rather than concentrated along internal load paths.

**Figure 1:**
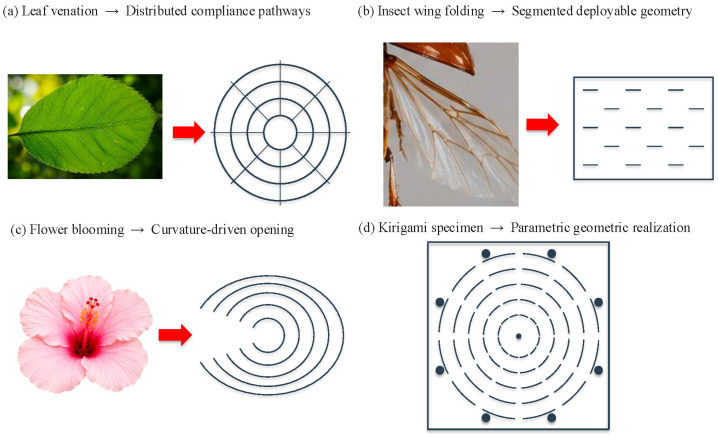
Biomimetic analogs of regime-dependent Kirigami deformation. Leaf venation, insect wing folding, and flower blooming are abstracted into distributed compliance, deployability, and curvature-driven opening. Schematic abstraction; panels are not to scale and do not depict literal biological structure.

Read together, these three biological systems share a common geometric logic: large, regime-dependent deformation is produced through the structured spatial distribution of compliance rather than through changes in constitutive material. Leaf venation distributes compliance through hierarchical pathways; insect wing folding programs it through cut and crease topology; flower blooming activates it through curvature modulation. The parametric Kirigami configurations examined here abstract these three principles into a single experimentally controlled design space, in which offset count and segmentation density correspond to distributed compliance pathways, boundary cut perimeter corresponds to deployable edge flexibility, and outer curvature corresponds to curvature-driven activation. The regime-dependent pattern of parameter effects reported in Section 5 is therefore expected on biological grounds: different loading conditions recruit different elements of the same geometric repertoire, just as different natural thin-sheet systems express different combinations of these mechanisms depending on functional context. Figure 1 summarizes this correspondence between the three natural analogs and the abstracted geometric principles realized in the Kirigami specimens.

At the level of shared structural feature category, both the natural exemplars and the tested Kirigami specimens organize compliance through openings and uncut bridges of order millimeters at specimen scales of order centimeters, with hierarchical or near-hierarchical spatial repetition. This places the tested design space within the same generic geometric regime as the cited biological systems—not as an exact numerical equivalence, but as a functionally comparable abstraction in which large reversible deformation arises from the spatial organization of cuts, ligaments, and boundaries rather than from changes in constitutive material. The empirical work in Section 4, Section 5 and Section 6 evaluates how parameters within this abstraction govern elastic displacement under two contrasting loading regimes.

### 2.6. Research Gaps and Operationalization

Three limitations in the literature motivate the present study. First, the role of individual geometric parameters in structuring elastic displacement has not been systematically differentiated within a shared parametric design space. Second, the conditionality of parameter influence across distinct loading regimes remains insufficiently tested. Third, the translation of empirical findings into bio-inspired parameter-selection guidance remains underdeveloped: prior studies document deformation outcomes, but provide limited regime-conditional governance for pre-fabrication geometric configuration. Computational inverse-design approaches have addressed related shape-programming problems [35,36,37], but these typically presuppose known target geometries rather than governing the selection of geometric parameters under regime-specific conditions. An empirically grounded classification of parameter roles across loading regimes, formulated as pre-fabrication design guidance, has not been established.

The present study addresses these limitations through a dual-regime evaluation of parametric Kirigami specimens, followed by threshold-based parameter classification, derivation of Parameter-Specific Design Implications, and their integration into a Bio-Inspired Regime-Dependent Parameter Selection in Parametric Kirigami. The contribution of the present study lies not only in identifying which geometric parameters exhibit regime-dependent influence within the tested space, but in translating that conditionality into actionable pre-fabrication guidance: a structured procedure that enables a designer to configure elastic displacement through conditional reasoning about parameter hierarchy, loading activation, and dependency resolution, prior to and independently of iterative fabrication.

## 3. Research Questions

This study examines how geometric parameters function as design variables in structuring elastic displacement in parametric Kirigami, and how their influence varies under different loading conditions. Elastic displacement is treated as a performance outcome, and geometric parameters are evaluated based on their capacity to produce consistent and directionally stable effects within a defined parametric design space. This enables differentiation between parameters that actively structure deformation behavior and those that remain Non-operative within the tested conditions.

Three research questions guide the analysis.

*Research Question 1 (Q1): Design Variables*

Which geometric parameters reliably structure elastic displacement within a bounded parametric Kirigami design space, and to what extent does their relative influence differ?

This question establishes the role of individual parameters as design variables, distinguishing those that reliably influence displacement from those that primarily organize geometric form without consistent behavioral effect.

*Research Question 2 (Q2): Regime Dependency*

To what extent is the influence of geometric parameters on elastic displacement stable across loading regimes, or conditionally dependent on deformation context?

By comparing tensile (pull) and gravity-drape loading, this question examines whether parameter–displacement associations are stable across regimes or contingent on loading condition, and whether parameters maintain or shift their roles across regimes.

*Research Question 3 (Q3): Bio-Inspired Parameter Selection*

Under what conditions, and for which geometric parameters, do empirically observed parameter–displacement associations support pre-fabrication parameter selection within the tested design space?

This question addresses the need for a structured classification that translates empirical parameter effects into actionable distinctions, enabling consistent reasoning about which variables should be prioritized, constrained, or treated as secondary in bio-inspired, regime-dependent parameter selection.

## 4. Research Design

**Figure 2:**
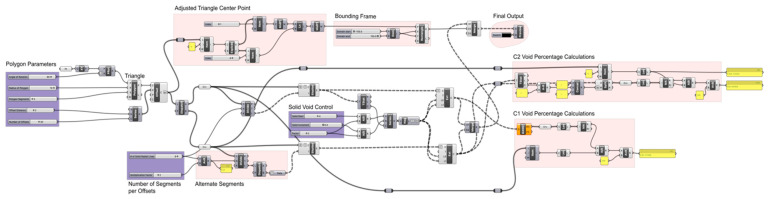
Parametric Grasshopper definition illustrating the generative logic of Bio-inspired Kirigami geometries across multiple base shapes, with all variables governing pattern formation.

A parametric design environment is established in which 25 geometric variables are systematically encoded, fabricated, and evaluated under controlled conditions to construct a governance-based understanding of elastic displacement in Kirigami systems, as presented in Figure 2. The method is structured to expose how geometric configurations produce condition-sensitive deformation behavior, enabling differentiation between parameters that reliably structure displacement and those that do not.

### 4.1. Specimen Geometry and Parametric Construction

Specimens are generated using parametric Grasshopper models within Rhinoceros 3D (McNeel & Associates), where all geometric parameters—offset count, offset spacing, segmentation, curvature radii, and boundary metrics—are defined algorithmically, as presented in Figure 2. This formulation treats geometry as an explicit design variable set, enabling controlled variation across a bounded design space.

Geometric outputs are transferred directly to fabrication through a CAD/CAM workflow, maintaining dimensional equivalence between computational definition and physical realization. Cutting is performed using a laser cutter equipped with a 150 W CO_2_ tube, operating at 18% to 23% of maximum power and a cutting speed of 50 mm/s. These parameters ensure consistent through-cuts across all three thermoplastic substrates without thermal distortion. The geometric center of each specimen is defined within the digital model and materialized as a 0.8 mm-centered hole to maintain consistent registration during measurement.

All specimens share uniform outer dimensions of 267 mm × 267 mm and are constructed through a concentric offset logic. The inner curve (C1) initializes the pattern, while additional curves are generated at uniform intervals to produce N parallel offsets within the outer boundary (C2). Five shape categories are included (circle, square, triangle, pentagon, hexagon), with circle and triangle most densely represented (*n* = 9 each). Pentagon and hexagon (*n* = 1 each) are retained descriptively and excluded from inferential analysis.

Three commercially sourced thermoplastic substrates are used: Clear (0.30 mm), Ruled (0.50 mm), and Grid (0.44 mm), differentiated by measured thickness and surface structure. Chemical identification of the three substrates was carried out at the Alfaisal Nanotechnology Research Lab using Fourier-transform infrared spectroscopy (FTIR), differential scanning calorimetry (DSC), and X-ray diffraction (XRD). The Cross (grid) and Lines (ruled) sheets exhibited characteristic polyethylene FTIR peaks, a melting endotherm at 125 °C in DSC, and sharp crystalline polyethylene reflections in XRD, and are identified as polyethylene (PE). The Clear (transparent) sheet exhibited ester-related FTIR bands at approximately 1730 cm^−1^ and 1250 cm^−1^, a lower melting transition at 105 °C in DSC, and a broader XRD profile consistent with reduced crystallinity, and is identified as ethylene-vinyl acetate copolymer (EVA). Calibrated constitutive properties (modulus, yield stress) were not measured in the present study, so material effects are interpreted at the level of substrate identity and thickness rather than through quantitative mechanical constants. The unit of analysis is therefore defined at the level of geometric configuration, with material treated as a secondary modulator [38].

**Figure 3:**
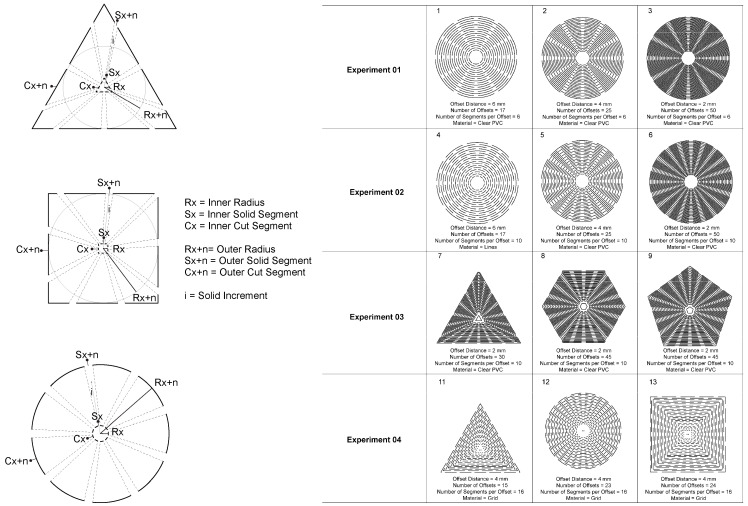
(**Left**) Schematic definition of inner/outer radii, solid and cut segments, and solid increment. (**Right**) Parametrically generated Kirigami variations across multiple geometries, showing the effect of offset, subdivision, and segmentation on pattern formation.

The experimental parameter set is organized into four functional categories (Table 1): independent geometric variables (IVs), dependent variables (DVs), boundary metrics (BMs), and categorical covariates (CCs). The IVs—offset distance, Number of Offsets, Segments per Offset, Solid Start Length (Sx), and solid increment (i)—define the generative design space, specifying the structural conditions under which elastic behavior emerges. Each parameter encodes a distinct aspect of geometric organization: offset count establishes the multiplicity of deformation pathways, segmentation regulates continuity along cut interfaces, and spacing parameters distribute material between adjacent curves. As shown in Figure 3 (left), Sx defines the initial uncut segment at the innermost curve, while i governs its incremental extension across successive offsets, together structuring the gradient of material continuity from center to boundary. The inner and outer radii (C1, C2) delimit the spatial field of the pattern, while the C2 perimeter and the C2 void perimeter act as derived boundary metrics quantifying the distribution of cut and solid along the outer edge. Within this formulation, the IVs correspond to Structure (S), while elastic displacement under tensile pull and gravity-drape regimes (Eval_ElasticityPullmm, Eval_ElasticityDropmm) constitutes Behavior (B), with the S → B mapping conditionally mediated by loading regime rather than fixed in geometry alone. Shape and material are therefore treated as categorical covariates that modulate the expression of deformation without redefining its geometric basis. Figure 3 (right) makes this relation explicit, showing how controlled variation in offset architecture, segmentation, and boundary definition produces distinct structural configurations from a shared generative logic, such that parameterization does not prescribe form directly, but structures the conditions under which deformation becomes mechanically admissible.

**Table 1:** Experimental variables, geometric notation, and analytical roles.

Variable (Dataset)	Definition	Role	Regime
Eval_ElasticityPullmm	Maximum vertical displacement under applied tensile loading	DV	Pull
Eval_ElasticityDropmm	Vertical displacement at the specimen center under gravity	DV	Gravity-drape
OffsetDistancemm	Perpendicular spacing between successive parallel curves	IV	Both
NumberofOffsets	Total number of parallel curves (inner to outer boundary)	IV	Both
NumberofSegmentsperOffset	Number of laser-cut segments per curve	IV	Both
SolidStartLengthmm (Sx)	Length of uncut segment on the inner curve	IV	Both
SolidIncrementmm (i)	Incremental increase in uncut segment length across offsets	IV	Both
C1Radiusmm (Rx)	Radius of the inner curve (R1)	IV	Both
C2Radiusmm (Rx + n)	Radius of the outermost curve (R2)	IV	Both
C2Perimeter	Total perimeter of the outermost curve	BM	Both
C2VoidPerimeter	Total length of cut segments along the outermost curve	BM	Both
Shape	Categorical boundary geometry (Circle/Square/Triangle/Pentagon/Hexagon)	CC	Both
Material	Plastic sheet type (Clear/Ruled/Grid); thick: 0.30, 0.50, 0.44 mm	CC	Both

Note. IV: independent geometric; DV: dependent variable; BM: boundary metric; CC: categorical covariate.

### 4.2. Mounting Configuration and Boundary Conditions

**Figure 4:**
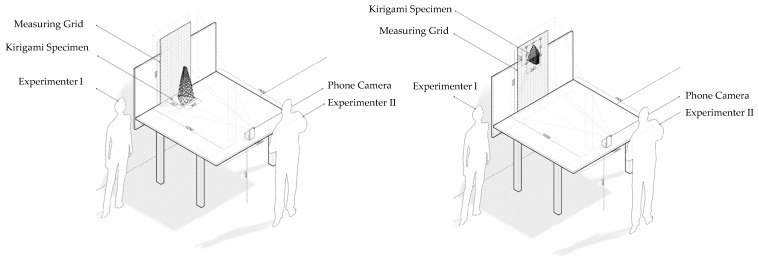
Kirigami specimen mounting and geometric notation. Left: pull-test attachment at geometric center. Right: specimen configuration for gravity-drape testing.

All specimens were mounted using an eight-point fixation protocol, with equidistant boundary attachments at 45° intervals (0–315°), establishing a controlled perimeter constraint that suppresses rigid-body motion while preserving internal deformation capacity. Boundary conditions were held constant across all tests to isolate the effects of geometric parameters. As shown in Figure 4, specimens were positioned vertically against a calibrated measuring grid serving as a fixed spatial reference for displacement capture, recorded via a perpendicular camera to minimize parallax error. Two distinct loading regimes were implemented. In the pull-test configuration (left), force is applied at the geometric center, intended to concentrate tensile loading across the interior offset architecture and produce displacement directed toward the constrained boundary; this configuration is designed to isolate the contribution of internal geometric structure under active loading. In contrast, the gravity-drape configuration (right) relies solely on self-weight, producing deformation dominated by boundary bending and distributed gravitational load rather than concentrated interior loading. These configurations represent fundamentally different activation conditions rather than variations in a single test: the former probes force-induced kinematic response, while the latter reveals deformation under gravitational loading. Maintaining identical boundary constraints across both regimes ensures that observed differences in elastic behavior arise from the interaction between geometry and loading condition, rather than from experimental variability.

### 4.3. Operationalizing Elasticity for Design Analysis

Elastic response is measured as absolute vertical displacement (mm), defined as the maximum reversible geometric deformation achieved prior to permanent structural change. Throughout this paper, “elastic,” “elastic displacement,” and “elastic capacity” refer to macroscopic kinematic behavior rather than constitutive elastic modulus or stored strain energy. Reversibility is defined by a recovery criterion of within 5% of the original planar diagonal after a two-minute stabilization period.

Within this formulation, elastic displacement functions as a design-relevant outcome through which the influence of geometric variables can be evaluated. Pull elastic displacement ranges from 40 to 950 mm (mean = 424.96 mm, SD = 240.01 mm), while gravity-drape displacement ranges from −8.0 to 797.5 mm (mean = 68.22 mm, SD = 166.81 mm).

### 4.4. Loading Regimes, Measurement Protocol, and Trial Validity

**Figure 5:**
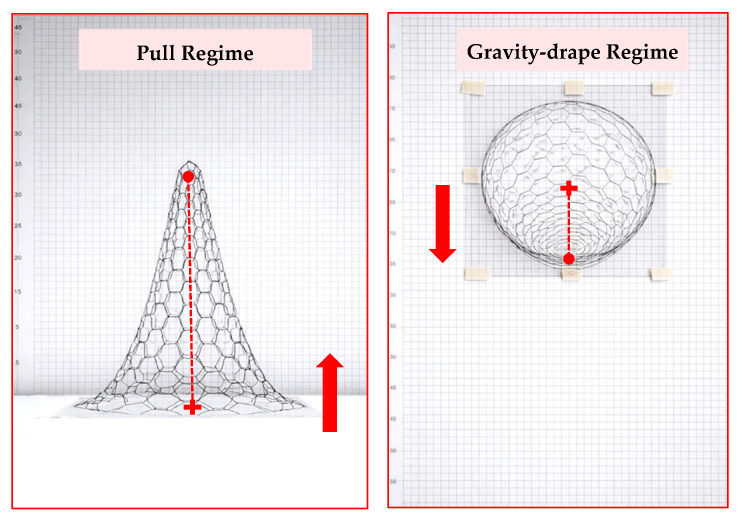
Kirigami elastic displacement under pull and gravity-drape regimes, illustrating contrasting displacement behaviors and geometric adaptation (Note: both photos are enhanced in Adobe Photoshop 27.4 to show Kirigami lines).

Two loading regimes are defined to expose distinct deformation logics within the same geometric configuration, as presented in Figure 5. Pull loading introduces a concentrated tensile force at the specimen center, producing a distributed geometric response across the offset architecture. Gravity-drape loading applies distributed self-weight, engaging large-scale bending and boundary-driven deformation.

Under pull loading, a centrally attached hook is drawn vertically downward using quasi-static progressive displacement by a single operator across all 25 specimens in a single session, eliminating inter-session variability. Displacement is recorded against a calibrated measurement grid by a second experimenter and verified through two simultaneous photographic readings taken from a standardized distance of 1250 mm using a fixed mobile camera. The three measurements are averaged, yielding an estimated precision of ±0.5–1.0 mm.

Termination of pull loading requires simultaneous satisfaction of two conditions: (1) a visual cue, defined as the specimen leaving the plane of the measurement grid, and (2) a tactile cue, defined as increased resistance indicating engagement beyond the elastic plateau. Measurement is taken at the onset of this transition to ensure capture of maximum elastic displacement.

Under gravity-drape loading, specimens are suspended from a fixed height of 1782 mm from floor level (950 mm above the table surface) and held until oscillation ceases. Elastic displacement is measured as the vertical displacement of the geometric center relative to a horizontal reference plane, with downward deformation recorded as positive and upward anti-clastic deformation as negative, measured 30 s after attachment.

All specimens are tested sequentially under both regimes (pull followed by gravity-drape). Recovery validity requires a return to within 5% of the original planar diagonal after two minutes. Of the initial 34 specimens, seven fail this criterion. Two additional Grid specimens are excluded following combined acoustic–visual failure, indicating fracture at the cut-tip hinge zones. The final dataset (*N* = 25) satisfies all validity conditions.

### 4.5. Analytical Strategy and Parameter Classification

The Kirigami geometry is treated as a coupled parametric design space in which multiple variables co-determine elastic response. The study is framed as an exploratory screening of geometric parameters: with *N* = 25 specimens covering 18 simultaneous parameter–displacement tests, Bonferroni-corrected statistical power for detecting |r| ≈ 0.40 at α/18 = 0.0028 is approximately 0.15 and at α = 0.05 is approximately 0.51. The sample is therefore sized to identify the strongest, threshold-stable parameter–displacement associations rather than to test moderate effects; weak or moderate associations identified here are treated as provisional and require confirmatory studies with *N* ≥ 60 specimens (the minimum sample size at which Bonferroni-corrected power for |r| = 0.40 reaches the conventional 0.80 threshold). This screening framing is the basis on which the three Bonferroni-robust pull-regime associations are interpreted as primary findings, and on which the gravity-drape associations are reported as suggestive rather than confirmatory. Regime-separated bivariate Pearson correlations are used to evaluate the relationship between individual geometric parameters and elastic displacement, allowing each parameter’s influence to be examined independently. Across all analyses, the bivariate Pearson r—and its shape-controlled partial counterpart—is the primary metric for ranking parameter influence and assigning governance tiers; R^2^ and partial η^2^ are reported only as supporting effect-size descriptors of variance explained and do not, by themselves, determine classification.

Pull and gravity-drape regimes function as complementary analytical conditions. Pull loading mobilizes distributed deformation pathways through concentrated energy input, while gravity-drape loading engages lower-energy, globally distributed deformation. Therefore, differences in elastic response across regimes reflect regime-dependent variation in parameter associations rather than differences in geometry itself.

To translate correlation magnitudes into design-relevant categories, parameters are classified into three tiers: Governing (|r| ≥ 0.40, *p* < 0.05); Modulating (|r| ≥ 0.30, with directional consistency); Non-operative (|r| < 0.30 within the tested design space).

Governance classification is based on the α = 0.05 threshold. Bonferroni correction (α/18 = 0.0028) is applied as a complementary criterion to differentiate Bonferroni-robust from non-Bonferroni-robust associations within the governing tier; it does not alter classification itself. In cases where bivariate classification falls near the governance threshold, shape-controlled partial correlations (Section 6.2) are used to assess whether the classification survives control for boundary geometry; where the partial coefficient falls below the threshold, the classification is revised accordingly and footnoted in the governance tables.

The governance threshold (|r| ≥ 0.40) is defined through empirical recalibration within the tested design space, representing the lowest value at which classification stability is preserved for dominant parameters while excluding marginal and unstable associations. Cohen’s benchmarks are treated as fallback norms [39,40], while empirical recalibration suggests that r = 0.30 already represents a large effect in applied research [41]. At |r| ≥ 0.40, a parameter explains at least 16% of the variance in elastic displacement, constituting a practically meaningful influence for design.

The selection of this threshold is further evaluated through a threshold robustness analysis (Table 2), which examines the stability of parameter classification under systematic variation in the decision threshold (|r| = 0.30–0.45). The results indicate that the principal governance signals—Number of Offsets (r = 0.807), Segments per Offset (r = −0.603), and C2 Void Perimeter (r = 0.621)—remain classified as Governing across all tested thresholds, indicating classification consistency within the tested design space. In contrast, parameters such as C2 Perimeter and solid increment exhibit classification drift as thresholds increase, transitioning between Governing and Modulating classifications or falling below the adopted governance threshold (Table 2). This instability indicates that their apparent influence is sensitive to threshold selection and, therefore, less reliable as design drivers.

The adopted threshold (|r| ≥ 0.40) represents the lowest value at which classification stability is preserved for the dominant parameters while excluding marginal and unstable associations. Lower thresholds admit parameters whose roles are not invariant, while higher thresholds begin to exclude parameters that remain structurally consistent within the design space. The threshold thus balances sensitivity and stability, preserving the minimal invariant structure of geometric influence.

Bonferroni correction (α/18 = 0.0028) is applied as a complementary criterion to assess evidential robustness under multiple testing (Table 4). This correction does not determine governance classification, but distinguishes statistically robust from non-Bonferroni-robust associations. The three Bonferroni-significant relationships—Number of Offsets, Segments per Offset, and C2 Void Perimeter under pull loading—coincide with the parameters exhibiting threshold-invariant classification, reinforcing their status as primary structural drivers of elastic displacement.

With *N* = 25, statistical power is approximately 0.51 for detecting |r| = 0.40 (α = 0.05, two-tailed), indicating potential Type II error for moderate effects. At the Bonferroni-corrected threshold (α = 0.0028), power to detect |r| = 0.40 is substantially lower (≈0.15); the three Bonferroni-robust associations (|r| ≥ 0.60) exceed the minimum detectable effect at this corrected threshold (|r| ≈ 0.69 at 80% power), supporting their inferential priority. Their stability across thresholds further reinforces their status as primary structural drivers. These results are therefore treated as structurally stable signals within the tested design space and as working hypotheses for confirmatory evaluation in larger samples (*N* ≥ 60).

### 4.6. Managing Geometric Coupling, Multicollinearity, and Curvature

The parametric construction logic introduces structural coupling among geometric variables, producing strong interdependencies that limit the reliability of multivariate regression. The most pronounced relationships occur between offset distance and solid increment (r = 0.905, *p* < 0.001) and between C1 and C2 radii (r = 0.858, *p* < 0.001), reflecting shared generative rules rather than independent design decisions. To prevent dependent parameters from being treated as independent controls, pairs with |r| ≥ 0.80 are designated a single coupled design axis and are not varied independently: Offset Distance–Solid Increment (r = 0.905), C1–C2 Radius (r = 0.858), and C2 Perimeter–C2 Void Perimeter (r = 0.889). A principal-component analysis of the coupled predictor block (Appendix A.6) confirms that these variables collapse onto a small number of latent geometric axes, and parameter selection operates on these axes rather than on the individual coupled variables.

Variance inflation factor (VIF) analysis confirms severe multicollinearity, with five predictors exceeding VIF = 10 (C2 Radius = 31.805; Offset Distance = 25.466; Number of Offsets = 22.336; C1 Radius = 14.824; Solid Increment = 10.410) and a condition index of 85.859. Under these conditions, regression coefficients become unstable and difficult to interpret [42,43]. Full collinearity diagnostics, including tolerance and variance inflation factors for all predictors, are reported in Appendix A.1.

The correlational screening approach provides a transparent basis for evaluating parameter influence prior to multivariate modeling. This allows each parameter’s relationship to elastic displacement to be examined directly, preserving interpretability within a coupled design space. The curvature parameters (C1Radiusmm, C2Radiusmm) are evaluated using nonlinear regression models (quadratic, cubic, piecewise-linear) to assess whether their influence emerges through nonlinear relationships. These models test whether curvature functions as a governing parameter or as a geometric organizer without direct mechanical consequence. Results are reported in Section 5.1.3.

## 5. Results

### 5.1. Parameter Effects

Elastic displacement varies systematically across the Kirigami design space under both pull and gravity-drape loading. Table 3 reports regime-separated Pearson correlations between elastic displacement and each geometric parameter. Across the tested parameter ranges, three geometric parameter clusters show the strongest associations with elastic displacement: the number of offset layers, the segmentation and offset spacing that structure hinge-like geometric configurations, and the total boundary cut length. Other descriptors primarily organize scale or pattern distribution and do not independently structure elastic displacement within the tested design space.

#### 5.1.1. Offset Architecture

The strongest association in Table 3 occurs between pull elastic displacement and the number of offsets (r = 0.807, *p* < 0.001), which remains Bonferroni-significant under multiple-testing correction (Table 4). This relationship accounts for approximately 65% of the variance in pull displacement (r^2^ = 0.651), suggesting that pull elastic displacement may be structured primarily by the number of offset layers available to participate in deformation. This pattern is consistent with the interpretation that each additional offset curve may introduce additional deformation pathways distributed through the specimen interior; the underlying mechanism was not directly instrumented in the present study. The same parameter shows a positive but attenuated association under gravity-drape loading (r = 0.411, *p* = 0.041), which does not survive Bonferroni correction (Table 4), indicating reduced evidential stability under passive loading conditions.

Offset distance exhibits a related but inverse pattern. Under pull loading, it is negatively associated with elastic displacement (r = −0.475, *p* = 0.017), suggesting that wider offset spacing may reduce the number of deformation-participating elements available within the pattern; this interpretation is consistent with the observed correlation (r = −0.475) but is not corroborated by instrumented measurement and does not survive Bonferroni correction (Table 4); therefore, it lacks evidential robustness. Under gravity-drape loading, the parameter shows no meaningful association (r = −0.106, *p* = 0.613). Offset distance is also strongly coupled to solid increment (r = 0.905), indicating that wider spacing necessarily increases the uncut material between offsets; within the present parametric structure, these effects cannot be independently resolved through bivariate analysis.

Segmentation density, defined as the number of laser-cut segments per offset curve, presents the second Bonferroni-robust governance signal under pull loading (r = −0.603, *p* = 0.001), which remains significant after correction (Table 4) and accounts for approximately 36% of variance (r^2^ = 0.364). The negative association indicates that increasing segmentation reduces elastic displacement. One interpretation consistent with this association is that additional segmentation introduces more solid bridges along each offset curve, interrupting the continuity of deformation pathways and constraining hinge-like motion—an interpretation consistent with the mechanics of segmented compliant structures in the Kirigami literature [4,10], though not directly confirmed through mechanism imaging or force mapping in the present study. As segmentation density increases, the correlational pattern is consistent with a transition from a more compliant to a more constrained configuration. Under gravity-drape loading, the association remains directionally consistent (r = −0.378) but does not reach statistical significance (*p* = 0.062) and does not survive Bonferroni correction (Table 4), indicating a modulating effect that does not retain statistical robustness under Bonferroni correction.

**Table 4:** Bonferroni-corrected significance summary (α = 0.0028, *N* = 25).

Parameter	Pull r	Pull p	Gravity-Drape r	Gravity-Drape p	Pull Status	Gravity-Drape Status
★ Number of Offsets	0.807	<0.001 ✦	0.411	0.041	BR	NBR
Offset Distance	−0.475	0.017	−0.106	0.613	NBR	NBR
★ Segments per Offset	−0.603	0.001 ✦	−0.378	0.062	BR	NBR
★ C2 Void Perimeter	0.621	<0.001 ✦	0.407	0.044	BR	NBR
C2 Perimeter	0.418	0.038	0.302	0.142	NBR	NBR
C2 Radius	0.377	0.064	0.414	0.040	NBR	NBR
C1 Radius	0.131	0.533	0.392	0.052	NBR	NBR
Solid Increment	−0.356	0.081	0.010	0.963	NBR	NBR
Solid Start Length	−0.023	0.914	0.176	0.401	NBR	NBR

★ = Bonferroni-robust; BR = Bonferroni-robust; NBR = non-Bonferroni-robust. ✦ *p* < 0.0028.

The solid pattern parameters—solid start length and solid increment—show limited evidence of independent influence within the tested design space. Both remain below the governing threshold under both regimes (|r| ≤ 0.356), and neither achieves Bonferroni significance (Table 4). Solid increment is strongly coupled with offset distance (r = 0.905), reinforcing its role as a dependent geometric consequence rather than an independent driver of deformation. Therefore, these parameters appear to structure the internal distribution of material rather than directly control global displacement magnitude.

#### 5.1.2. Boundary Geometry

Boundary metrics show the most stable cross-regime relationship with elastic displacement. The perimeter of cut segments along the outer boundary (C2VoidPerimeter) correlates positively with elastic deformation under both loading regimes (pull r = 0.621, *p* < 0.001; gravity-drape r = 0.407, *p* = 0.044), indicating a consistent geometric contribution across conditions, although the association is Bonferroni-robust under pull loading only and exhibits reduced evidential stability under gravity-drape.

A physical interpretation consistent with the cross-regime association is that boundary cut segments create free-edge bending compliance at the specimen perimeter, engaged under both loading conditions, because both pull and gravity-drape loading transmit deformation through the boundary to the fixed attachment points. This interpretation accounts for the observed cross-regime stability (pull r = 0.621; gravity-drape r = 0.407), though the underlying compliance mechanism was not directly measured in the present study.

By contrast, the total outer perimeter (C2Perimeter) shows only moderate associations with elastic displacement (pull r = 0.418, *p* = 0.038; gravity-drape r = 0.302, *p* = 0.142), placing it in the modulating rather than governing category. The comparison indicates that elastic displacement is associated less with total boundary length than with the proportion of that boundary that is cut. Because C2VoidPerimeter can be modified independently of specimen size, it meets the governance threshold across both regimes (|r| ≥ 0.40), with Bonferroni-robust evidence under pull and reduced evidential stability under gravity-drape.

#### 5.1.3. Curvature Parameters

Curvature parameters were examined to determine whether global geometric scale contributes independently to elastic displacement beyond the parameter associations established above. Table 3 shows moderate but unstable associations between curvature and elastic response. C2Radius exhibits a moderate positive correlation under gravity-drape loading (r = 0.414, *p* = 0.040) but only a marginal association under pull loading (r = 0.377, *p* = 0.064). C1Radius shows no meaningful relationship under pull loading (r = 0.131, *p* = 0.533) and only a borderline association under gravity-drape loading (r = 0.392, *p* = 0.052). These patterns suggest that curvature primarily organizes geometric scale rather than directly governing elastic displacement.

**Figure 6:**
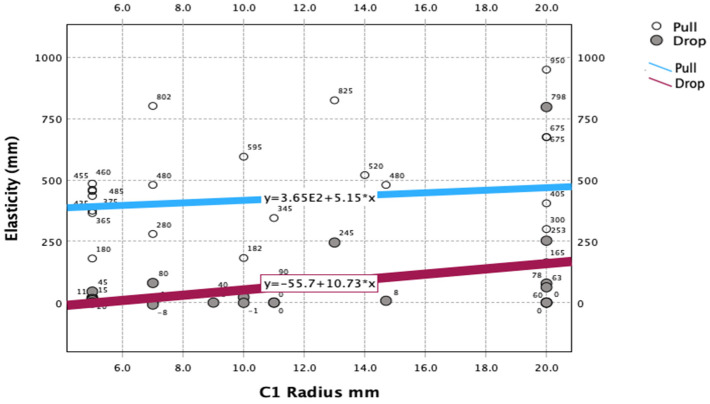
Scatter plots of elastic displacement against inner curvature radius (C1Radiusmm) under pull and gravity-drape loading. *N* = 25.

Inner Curvature (C1 Radius). The inner curvature radius (C1Radiusmm) occupies a structurally central position within the parametric construction logic, correlating strongly with offset distance (r = 0.641), solid increment (r = 0.675), and outer radius (C2Radiusmm; r = 0.858). Despite this geometric centrality, C1Radiusmm shows no meaningful association with elastic displacement under pull loading (r = 0.131, *p* = 0.533), and only a borderline association under gravity-drape loading (r = 0.392, *p* = 0.052)—neither survives Bonferroni correction (Table 4), placing it below the threshold for operative influence and therefore classifying it as Non-operative under both regimes once shape is controlled (r_part = 0.275, Table 13). This absence of stable association is evident in the scatter plots in Figure 6, which show no discernible trend under either loading condition.

To test whether a nonlinear curvature–deformation relationship was being obscured by linear correlation, quadratic, cubic, and piecewise-linear regression models were estimated, as presented in Table 5. Across all models and both loading regimes, explanatory power remains minimal (R^2^ = 0.018–0.048 under pull; R^2^ = 0.162–0.165 under gravity-drape), and no specification reaches statistical significance (all *p* > 0.14).

Taken together, the correlation (Table 3), distributional evidence (Figure 6), and nonlinear modeling results (Table 5) indicate that inner curvature functions as a geometric organizer rather than a mechanical driver of elastic displacement. Although C1Radiusmm structures the spatial arrangement of several other parameters within the offset construction logic, this organizational role does not translate into direct influence on deformation magnitude. Within the tested parameter ranges and after shape control, inner curvature does not demonstrate a stable or independent influence on elastic displacement and is therefore classified as Non-operative.

**Table 5:** Nonlinear regression models—C1 Radius (inner curvature). Elastic displacement as outcome variable. Pull and Gravity-drape regimes. *N* = 25.

Model	Pull R^2^	Pull Adj. R^2^	Pull SE	Pull F (df)	Pull p	GD R^2^	GD Adj. R^2^	GD SE	GD F (df)	GD p
Quadratic f(C1, C1^2^)	0.018	−0.071	248.43	0.200 (2, 22)	0.820	0.164	0.088	159.32	2.155 (2, 22)	0.140
Cubic f(C1, C1^2^, C1^3^)	0.048	−0.088	250.37	0.352 (3, 21)	0.788	0.165	0.046	162.96	1.382 (3, 21)	0.276
Piecewise k = 12 mm	0.018	−0.071	248.36	0.206 (2, 22)	0.815	0.162	0.086	159.49	2.127 (2, 22)	0.143

Note. OLS regression; two-tailed *p*-values. *N* = 25. Pull and Gravity-drape elastic displacement as outcome variables. C1 = inner curvature radius. All C1 nonlinear models are non-significant across both regimes (*p* > 0.14). C1 is classified as Non-operative in Pull and attenuates to Non-operative in Gravity-drape after shape control (see Table 13). GD = Gravity-drape.

**Figure 7:**
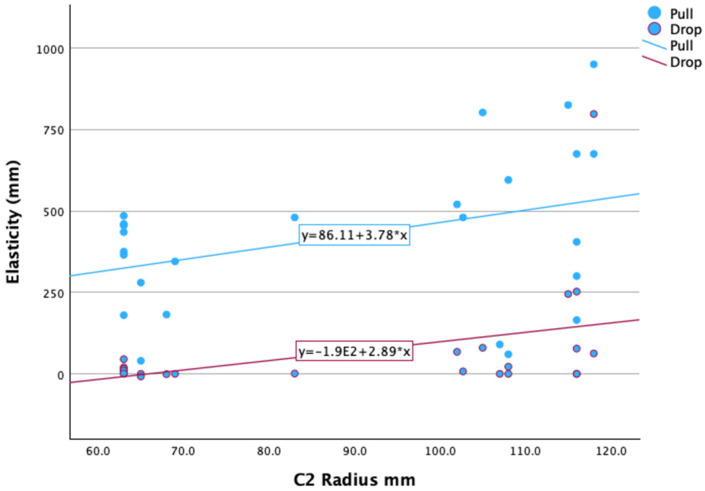
Scatter plots of elastic displacement against outer curvature radius (C2Radiusmm) under pull and gravity-drape loading. *N* = 25.

Outer Curvature (C2 Radius). In contrast to the inner radius, the outer boundary radius (C2Radiusmm) shows evidence of a regime-dependent relationship with elastic displacement. Linear correlation analysis (Table 3) indicates only a moderate and non-significant association under pull loading (r = 0.377, *p* = 0.064), but a statistically significant association under gravity-drape loading (r = 0.414, *p* = 0.040). This divergence is reflected in the scatter distributions (Figure 7), where no clear pattern emerges under pull, while a positive trend is visible under gravity-drape conditions. Because curvature effects may be nonlinear, the same regression specifications used for C1 were estimated for C2 (Table 6).

Under pull loading, none of the nonlinear models produce significant explanatory power (R^2^ = 0.142–0.163; all *p* ≥ 0.064), confirming that outer curvature does not independently govern elastic displacement when deformation is induced through tensile loading. In contrast, under gravity-drape loading, nonlinear models converge on a modest but consistent association. Quadratic, cubic, and piecewise specifications all reach conventional significance (adjusted R^2^ ≈ 0.222–0.229; *p* ≈ 0.022–0.040), with the cubic model yielding the highest adjusted R^2^ (0.229), although the improvement over the quadratic model is marginal (Δadj. R^2^ = 0.007).

Taken together, the correlation (Table 3), distributional evidence (Figure 7), and nonlinear modeling results (Table 6) indicate that outer curvature operates as a regime-dependent geometric influence. Under gravity-drape loading, deformation is governed by large-scale bending of the specimen envelope, making global curvature more consequential. Under pull loading, internal offset architecture parameters show stronger and more stable associations with elastic displacement, with boundary curvature exhibiting a comparatively marginal association. Given the small sample size (*N* = 25) and the absence of Bonferroni significance (α = 0.0028), the curvature effect is interpreted as a nonlinear but non-governing effect within the tested design space.

### 5.2. Regime Comparison

Elastic responses under pull and gravity-drape loading are positively correlated (r = 0.665, *p* < 0.001), indicating a shared geometric baseline of deformability. Approximately 44% of the variance in elastic displacement is common across regimes (r^2^ = 0.442), suggesting that certain structural characteristics of Kirigami geometry predispose specimens to deform more readily regardless of loading condition. At the same time, 56% of variance remains regime-specific, and the parameter profile differs systematically between pull and gravity-drape loading.

Two patterns follow from Table 3. First, pull loading produces the strongest governance effects overall. Number of Offsets (r = 0.807), Segments per Offset (r = −0.603), and C2VoidPerimeter (r = 0.621) all meet governing-level criteria under pull loading, with the first and third also showing positive but attenuated effects under gravity-drape conditions. Second, several parameters change status across regimes. Offset Distance is influential under pull (r = −0.475, *p* = 0.017) but Non-operative under gravity-drape (r = −0.106, *p* = 0.613). Segments per Offset remain directionally consistent across regimes but weaken from governing under pull to modulating under gravity-drape. C2Radius displays the inverse pattern, becoming more relevant under gravity-drape than under pull.

The contrast indicates that elastic displacement is not expressed uniformly across loading conditions. Pull loading corresponds to stronger associations with internal offset architecture parameters—offset count and segmentation density—whereas gravity-drape loading corresponds to greater relative influence of boundary geometry parameters. Therefore, the same geometric configuration yields systematically different parameter–displacement associations depending on loading condition.

### 5.3. Robustness

#### 5.3.1. Shape Typology

Shape typology was examined to determine whether observed parameter associations could be artifacts of boundary geometry. The dataset includes five shape categories (Circle *n* = 9, Triangle *n* = 9, Square *n* = 5, Pentagon *n* = 1, Hexagon *n* = 1). Pentagon and hexagon are retained descriptively but excluded from statistical inference due to insufficient sample size.

Correlation analysis indicates that no shape dummy variable reaches the governance criterion under either loading regime (|r| ≥ 0.40, *p* < 0.05), as shown in Table 7, indicating that boundary topology alone does not independently structure elastic displacement within the tested design space.

Some directional tendencies are nonetheless observable within the data (Table 7). Circular specimens show the strongest positive association with gravity-drape displacement (r = 0.304), consistent with the governing nonlinear curvature effect observed for C2Radiusmm, while triangular specimens exhibit negative associations under both regimes (pull r ≈ −0.243; gravity-drape r ≈ −0.261), suggesting that angular boundary geometries may introduce localized stiffness effects. However, these associations remain below the governance threshold and do not exhibit stability across regimes.

Accordingly, shape typology operates as a geometric descriptor of boundary form rather than a governing variable of elastic displacement within the present dataset.

#### 5.3.2. Shape-Controlled General Linear Model (GLM) Analysis

To examine whether the observed geometric effects are confounded by boundary shape, a general linear model (GLM) was estimated with shape as a fixed factor, and C2Perimeter included as a covariate to control for boundary scale (Table 11). The results indicate that shape does not exert a statistically significant effect on elastic displacement (F(4, 40) = 0.683, *p* = 0.608), and no shape × loading interaction is observed (F(4, 40) = 0.194, *p* = 0.940).

In contrast, loading regime remains the dominant source of variance (F(1, 40) = 17.610, *p* < 0.001), indicating that deformation behavior is primarily structured by activation condition rather than boundary typology. The inclusion of C2Perimeter accounts for size-related variation, isolating shape from scale effects within the model.

Taken together, these results provide no statistical evidence that the primary geometric effects identified in Table 3 are attributable to boundary shape within the tested dataset. The non-significant shape and shape × loading interaction effects are consistent with the interpretation that elastic displacement is governed by parameter-level geometric configuration rather than boundary typology; however, the limited sample size constrains the power to detect moderate shape effects, and this conclusion should be treated as provisionally supported pending confirmatory evaluation in larger factorial samples.

### 5.4. Secondary Modulators

#### 5.4.1. Material Type

Material type and shape were examined as secondary modulators—variables that may condition how geometric capacity is expressed under a given regime, but which do not independently structure elasticity once loading regime and key geometric descriptors are taken into account.

Material effects were evaluated with a two-way general linear model (Type III sums of squares), specifying material type, loading mode, and geometric covariates as fixed effects (Table 8). Specimen-level variance was negligible (σ^2^_specimen ≈ 0; ICC ≈ 0), indicating no residual clustering once covariates were controlled; full variance-component estimates and information criteria are reported in Appendix A.5. Within these limits, the model identifies a significant material main effect (F(2, 36) = 4.254, *p* = 0.022) alongside a dominant loading mode effect (F(1, 36) = 109.568, *p* < 0.001), while the material × mode interaction does not reach significance (F(2, 36) = 2.391, *p* = 0.106). These results are consistent with the interpretation that material type conditions elastic response without altering the regime-dependent structure of deformation.

A sensitivity analysis replacing C2Perimeter with C2VoidPerimeter as the boundary covariate confirms this structure, with the material main effect (F(2, 36) = 4.098, *p* = 0.025, partial η^2^ = 0.185) and loading mode effect (F(1, 36) = 112.237, *p* < 0.001, partial η^2^ = 0.757) preserved, and the interaction remaining non-significant (F(2, 36) = 2.449, *p* = 0.101), indicating that the material effect is not an artifact of boundary parameter specification (Appendix A.4).

Estimated marginal means (Table 9) reveal a regime-dependent material ordering. Under pull loading, Ruled exhibits the highest adjusted mean (540.6 mm), followed by Grid (514.6 mm) and Clear (272.2 mm). Under gravity-drape loading, Grid exhibits the highest displacement (260.9 mm), followed by Ruled (103.7 mm), while Clear produces a negative adjusted mean (−110.8 mm), indicating anti-clastic deformation upward. This reversal is consistent with the low self-weight of the thinnest substrate, where gravitational loading is insufficient to overcome boundary constraint, resulting in curvature inversion rather than downward sag.

Within the parameter selection procedure developed in Section 7, these findings position material as a Non-operative but performance-relevant variable within the present dataset—exerting a statistically detectable effect on displacement magnitude (F(2, 36) = 4.254, *p* = 0.022) without satisfying the governance threshold (|r| ≥ 0.40) that defines governing status within the classification system, rather than a governing design variable. Material selection does not define the deformation capacity of the system—that role is associated with primary geometric parameters within the present dataset—but modulates how that capacity is expressed under specific loading conditions. In this sense, material operates at the level of parameter refinement, comparable to secondary geometric variables: it adjusts the magnitude, direction, and stability of response without redefining the underlying deformation logic.

Accordingly, substrate selection should be treated as a conditional decision within the design sequence: applied after primary geometric configuration is established, and evaluated relative to the intended loading regime. Material choice therefore participates in the pre-fabrication parameter selection process not as a source of form, but as a regulator of performance expression within an already structured geometric system, within the bounds of the present dataset.

**Table 9:** Estimated marginal means of elasticity by material type and loading mode from the fixed-effects general linear model, adjusted for geometric covariates at observed means.

Material	Mode	Adjusted Mean (mm)	SE	95% CI
Clear	Pull	272.2	76.1	[117.7, 426.8]
Clear	Gravity-drape	−110.8	76.1	[−265.3, 43.8]
Ruled	Pull	540.6	89.9	[358.0, 723.3]
Ruled	Gravity-drape	103.7	89.9	[−78.9, 286.3]
Grid	Pull	514.6	64.6	[383.3, 645.9]
Grid	Gravity-drape	260.9	64.6	[129.6, 392.2]

#### 5.4.2. Shape

Shape effects were examined using two complementary analytical strategies: descriptive distribution (Table 10) and inferential modeling through a general linear model (GLM) controlling for boundary scale (Table 11). The dataset comprises balanced observations across loading regimes (*n* = 25 pull; *n* = 25 gravity-drape), with within-specimen correlation (r = 0.665, *p* < 0.001) motivating separation of descriptive and model-based interpretation.

Descriptive statistics (Table 10) indicate variation in mean elastic displacement across shape categories. Under pull loading, squares (495.4 mm) and circles (445.2 mm) exhibit higher mean displacement than triangles (348.9 mm), while under gravity-drape loading, circles show the highest mean response (134.6 mm), followed by squares (63.6 mm) and triangles (11.3 mm). Pentagon and hexagon categories are retained descriptively (*n* = 1 each) but excluded from inference. These distributions suggest potential shape-related differences; however, they do not establish independent shape effects due to confounding with boundary scale and geometric parameters.

**Table 10:** Sample distribution by shape and loading mode (*N* = 50 stacked).

Shape	N (Pull)	N (Gravity-Drape)	Total N	Mean Pull (SD)	Mean Gravity-Drape (SD)
Circle	9	9	18	445.2 (296.2)	134.6 (261.5)
Square	5	5	10	495.4 (321.6)	63.6 (107.6)
Triangle	9	9	18	348.9 (147.7)	11.3 (14.7)
Hexagon †	1	1	2	480.0 (—)	7.5 (—)
Pentagon †	1	1	2	520.0 (—)	67.5 (—)
Total	25	25	50	424.96 (240.0)	68.22 (166.8)

Note. † Pentagon (*n* = 1) and Hexagon (*n* = 1) are descriptive only; excluded from all inferential analyses. Shape category is not a continuous predictor; mean values reflect descriptive summary only and do not imply significant shape effects (see Table 11). Within-specimen correlation between Pull and Gravity-drape displacements: r = 0.665 (*p* < 0.001), motivating stacked-data GLM design for Table 11.

**Table 11:** General Linear Model (GLM): effects of shape and loading mode on elasticity, controlling for C2 perimeter (*N* = 50).

Effect	df	F	*p*	Partial η^2^	Interpretation
Corrected Model	9, 40	4.140	<0.001	0.482	Overall model significant
ElasticityMode (Pull vs. Gravity-drape)	1, 40	17.610	<0.001	0.306	Dominant: regime effect
Shape	4, 40	0.683	0.608	0.064	Non-significant
Shape × ElasticityMode	4, 40	0.194	0.940	0.019	Non-significant interaction
C2Perimeter (covariate)	—	—	—	—	Included for scale control

Model: R^2^ = 0.482; Adjusted R^2^ = 0.366.

Shape-controlled GLM analysis (Section 5.3.2; Table 11) confirms no significant independent shape effect on elastic displacement (F(4, 40) = 0.683, *p* = 0.608), with loading regime as the dominant variance source (F(1, 40) = 17.610, *p* < 0.001).

Accordingly, the apparent differences observed in Table 10 are attributable to covariation with boundary scale and geometric configuration rather than independent shape effects. Within the tested design space, shape does not satisfy the governance criterion and does not independently structure elastic displacement; its influence remains secondary to parameter-level geometric configuration.

## 6. Regime-Dependent Parameter Influence on Elastic Displacement

### 6.1. Parameter Governance Across the Design Space

Table 12 consolidates the classification of geometric parameters across pull and gravity-drape loading regimes, based on the classification criteria established in Section 4.5 and the empirical relationships identified in Section 5.1.1, including the distinction between Bonferroni-robust and non-Bonferroni-robust associations. Parameters are differentiated into governing, modulating, and Non-operative roles according to the magnitude and stability of their associations with elastic displacement.

Across the design space, elastic displacement is organized around three geometric parameter clusters that account for the predominant share of its variance: offset architecture, segmentation density, and boundary cut length. The Number of Offsets governs elastic displacement across both regimes, while Offset Distance governs deformation under pull loading only. Segmentation density governs under pull and modulates under gravity-drape loading. Boundary cut length, represented by C2VoidPerimeter, meets the governance threshold across both regimes (|r| ≥ 0.40), with Bonferroni-robust evidence under pull and reduced evidential stability under gravity-drape. Other parameters—C2Perimeter, C2Radius, and Solid Increment—exert secondary influence, while C1Radius and Solid Start Length do not demonstrate stable control within the tested design space. The two per-regime classification columns in Table 12—Pull Class. and Gravity-Drape Class., each assigning every parameter to a governing, modulating, or Non-operative tier—are intended as an at-a-glance categorical layer that allows designers and researchers to locate the dominant governing variables without parsing the full coefficient set, while the Regime Stability Pattern column and the tier labels in the preceding correlation tables serve the same orienting purpose.

**Table 12:** Parameter governance map across loading regimes. Classification, stability, and Bonferroni-corrected significance (α = 0.0028).

Parameter	Pull Class.	Gravity-Drape Class.	Pull Bonf.	Gravity-Drape Bonf.	Regime Stability Pattern
Number of Offsets (r = 0.807/0.411)	Governing	Governing	BR (*p* < 0.001)	NBR (*p* = 0.041)	✦ Stable—Governing both regimes
Offset Distance (r = −0.475/−0.106)	Governing	Non-operative	NBR (*p* = 0.017)	NBR (*p* = 0.613)	Pull-Exclusive
Segments per Offset (r = −0.603/−0.378)	Governing	Modulating	BR (*p* = 0.001)	NBR (*p* = 0.062)	Pull-Dominant
C2 Void Perimeter (r = 0.621/0.407)	Governing	Governing	BR (*p* < 0.001)	NBR (*p* = 0.044)	✦ Stable—Governing both regimes
C2 Perimeter (r = 0.418 †/0.302)	Modulating	Modulating	NBR (*p* = 0.038)	NBR (*p* = 0.142)	Modulating both regimes
C2 Radius (r = 0.377/NL)	Modulating	Governing (NL)	NBR (*p* = 0.064)	NBR (*p* = 0.022)	Modulating in Pull; Governing (NL) in Gravity-drape
C1 Radius (r = 0.131/0.392 †)	Non-operative	Non-operative	NBR (*p* = 0.533)	NBR (*p* = 0.052)	Non-operative in both regimes
Solid Increment (r = −0.356/0.010)	Modulating	Non-operative	NBR (*p* = 0.081)	NBR (*p* = 0.963)	Modulating in Pull; Non-operative in Gravity-drape
Solid Start Length (r = −0.023/0.176)	Non-operative	Non-operative	NBR (*p* = 0.914)	NBR (*p* = 0.401)	Non-operative in both regimes

BR = Bonferroni-robust; NBR = Non-Bonferroni-robust. ✦ Stable—Governing both regimes. Note. † Governance classification revised following shape-controlled partial correlation analysis. C2 Perimeter under Pull loading: the bivariate coefficient (r = 0.418) attenuates to r_part = 0.371 (*p* = 0.097) once shape category is controlled, falling below the Governing threshold; reclassified as Modulating. C1 Radius under Gravity-drape loading: bivariate Modulating status (r = 0.392) does not survive shape control (r_part = 0.275, *p* = 0.228); reclassified as Non-operative.

### 6.2. Governance Stability Under Shape Control

To assess whether parameter governance is contingent on boundary shape, shape-controlled partial correlations were computed (Table 13), comparing bivariate (r_biv) and shape-controlled (r_part) associations for each parameter across loading regimes. Shape category was dummy-coded using Circle as the reference group, with four binary predictors (Triangle, Square, Pentagon, Hexagon); partial correlations were computed by partialling out all four shape dummy variables simultaneously. Stability is evaluated in terms of governance classification, not coefficient invariance.

The results indicate that the principal governance signals remain stable under shape control. Under pull loading, the dominant parameters—Number of Offsets, Segments per Offset, and C2VoidPerimeter—retain or strengthen their associations (e.g., r = 0.807 → 0.825; r = −0.603 → −0.667; r = 0.621 → 0.633), consistent with the interpretation that these associations are not artifacts of boundary topology. C2Perimeter shows modest attenuation (r = 0.418 → 0.371), falling below the governing threshold but remaining within the modulating range, indicating limited sensitivity to shape.

Under gravity-drape loading, similar patterns are observed. C2VoidPerimeter strengthens under shape control (r = 0.407 → 0.480), while Number of Offsets (r = 0.411 → 0.463) and C2Perimeter (r = 0.302 → 0.324) increase modestly without altering their classification. C1Radius exhibits attenuation (r = 0.392 → 0.275), consistent with its classification as Non-operative once shape is controlled. Other parameters remain stable, with no shifts in governance category.

Taken together, the results in Table 13 demonstrate that the principal geometric drivers of elastic displacement are largely independent of boundary shape. Shape does not act as a confounding variable in the identification of governing parameters, and the governance structure defined in Table 12 remains invariant under shape-controlled conditions, consistent with the GLM results in Section 5.3.2.

**Table 13:** Bivariate and shape-controlled partial Pearson correlations between geometric parameters and elastic displacement. Pull and gravity-drape regimes. *N* = 25.

Parameter	Pull r_biv	Pull r_Part	Pull Stability	GD r_biv	GD r_Part	GD Stability
Number of Offsets	0.807	0.825	→ Stable	0.411	0.463	→ Stable
Offset Distance	−0.475	−0.571	↑ Strengthened	−0.106	−0.286	→ Stable
Segments per Offset	−0.603	−0.667	↑ Strengthened	−0.378	−0.384	→ Stable
C2 Void Perimeter	0.621	0.633	→ Stable	0.407	0.480	↑ Strengthened
C2 Perimeter	0.418	0.371	↓ Attenuated	0.302	0.324	→ Stable
Solid Increment	−0.356	−0.471	↑ Strengthened	0.010	−0.168	→ Stable
C2 Radius	0.377	0.385	→ Stable	†	(0.328)	—
C1 Radius	0.131	0.013	→ Stable	0.392	0.275	↓ Attenuated
Solid Start Length	−0.023	0.038	→ Stable	0.176	0.266	→ Stable

GD = gravity-drape. → Stable; ↑ Strengthened; and, ↓ Attenuated. Note. Stability denotes consistency in governance classification rather than invariance of coefficient magnitude. † Indicates attenuation under shape-controlled partial correlation, resulting in reclassification where applicable.

### 6.3. Conditional Governance Across Loading Regimes

Elastic displacement, within the present dataset, does not arise from geometric configuration alone but from the conditional interaction between geometric structure and loading regime, as established through the classification parameter selection procedure (Section 4.5) and empirical results (Table 3 and Table 4; Section 5.1, Section 5.2 and Section 5.3) and consolidated in Table 12 and Table 13. These relationships do not represent independent parameter effects, but a conditionally structured hierarchy of geometric influence.

This hierarchy is not static; it emerges differently depending on the loading conditions under which deformation is realized. Within this formulation, geometric parameters exhibit influence that is contingent on loading regime, and design is not the direct specification of form but the anticipation of the conditions under which specific geometric configurations are behaviorally consequential. Therefore, geometric configuration and loading regime operate as co-dependent factors, with loading regime acting as the activating context that conditions which geometric parameters determine elastic behavior, rather than as a variable symmetric with geometry.

Pull-dominant governance (active loading): Number of Offsets is governing under pull (r = 0.807, Bonferroni-robust); attenuated under gravity-drape (r = 0.411, not Bonferroni-robust). Offset Distance shows a negative association under pull (r = −0.475, *p* = 0.017); Non-operative under gravity-drape. Segments per Offset is governing under pull (r = −0.603, Bonferroni-robust); modulating under gravity-drape (r = −0.378).

Gravity-drape nonlinear association (passive loading): C2 Radius exhibits a nonlinear association under gravity-drape (adj. R^2^ = 0.229, *p* = 0.022), interpreted as a nonlinear but non-governing effect within the tested design space.

Cross-regime governance: C2 Void Perimeter meets the governance threshold under both regimes (based on |r| ≥ 0.40 threshold), with Bonferroni-robust evidence under pull (r = 0.621) and reduced evidential stability under gravity-drape (r = 0.407).

Cross-regime modulation: C2 Perimeter is modulating under both regimes, with attenuation under shape control.

Non-operative parameters: C1 Radius is Non-operative under both regimes after shape control. Solid Increment and Solid Start Length structure pattern configuration without a consistent influence on elastic displacement.

Elastic displacement therefore emerges not as an intrinsic property of geometry, but as a regime-dependent realization of latent geometric capacity. Geometry supports a range of possible deformation outcomes within the tested design space, while the loading condition corresponds to the differential realization of those latent capacities as observable displacement.

### 6.4. From Parameter Governance to Design Reasoning

The preceding analyses suggest that elastic displacement, within the tested Kirigami design space, is not uniformly structured by all geometric variables, but is instead governed by a subset of parameters whose influence varies across loading regimes. Number of Offsets and C2VoidPerimeter demonstrate the most consistent associations across conditions, while offset distance and segmentation exhibit regime-dependent effects, and curvature parameters remain conditional or Non-operative within the tested ranges.

Crucially, these findings not only differentiate parameters—they indicate a structured design space in which geometric variables occupy distinct functional roles relative to elastic behavior. This structure is not descriptive but operational: it indicates which parameters can be relied upon to influence displacement, under what conditions, and with what degree of stability.

At this point, the analysis reaches a limit if treated purely as a statistical classification. Correlation alone does not constitute design knowledge unless it can be mobilized for decision-making. Therefore, the question shifts from what governs behavior to how such governance can be used to guide design. In this sense, within the tested design space, design does not so much select geometry as select the conditions under which geometry becomes operative.

This transition requires translating parameter-level associations into condition-sensitive design reasoning. Within this formulation, geometric parameters are no longer treated as independent descriptors but as decision variables whose influence is contingent on the loading regime. The distinction between Governing, Modulating, and Non-operative parameters provides the basis for such translation, enabling the formulation of conditional relationships between geometry and expected displacement.

The following section develops this translation as a parameter selection procedure, in which empirical findings are reformulated as latent capacity, regime activation, and Parameter-Specific Design Implications that support design-stage geometric configuration within the tested design space.

## 7. Translating Empirical Associations into Parameter-Selection Guidance

The findings do not merely classify geometric parameters—they reframe elastic displacement as a conditionally governed design outcome whose realization depends on the coordinated interaction between geometric configuration and loading regime. Within this formulation, parameters operate at differentiated levels of governance—Governing, Modulating, or Non-operative—with effects that are not intrinsic properties of form but are conditionally realized through the specific regime under which deformation is engaged. Geometric configuration, in this sense, structures a field of latent deformation potentials; the loading regime determines which of those potentials are expressed as observable displacement.

Yet classification and regime comparison remain analytically incomplete unless translated into a form that directly supports design action. Identifying which parameters matter is necessary but not sufficient: designers must also determine how to prioritize them, how to resolve dependencies among coupled variables, and how to combine governing and modulating effects relative to a specified performance intent. Without such a structure, each design instance requires independent reinterpretation of empirical results, limiting the practical transferability of the parameter selection procedure.

This section addresses that gap by translating parameter associations into a decision-oriented procedure for adaptive geometric configuration (Q3). The parameter selection procedure does not propose universal rules; rather, it structures conditional design reasoning by linking performance intent, loading regime, and parameter hierarchy into a coherent and repeatable sequence of decisions—enabling elastic displacement to be approached as a configurable outcome prior to fabrication rather than a discovered consequence of it.

### 7.1. Geometry as a Conditional Carrier of Elastic Displacement

The conditional regime-dependence documented in Section 5 supports treating Kirigami geometry as exhibiting latent elastic capacity: a set of deformation potentials that are differentially expressed under specific loading conditions. Geometry alone does not determine behavior; rather, the observed associations are contingent on both structural configuration and loading regime.

This can be interpreted as a conditional mapping: Structure × Loading → Behavior (S × L → B), rather than a direct structural determination of behavior. The loading regime acts as an activating condition that selectively engages portions of the geometric deformation space.

From a design perspective, this implies that geometric decisions cannot be interpreted independently of anticipated loading conditions.

### 7.2. Effect of Loading Regime on Parameter Hierarchy

The contrast between pull and gravity-drape regimes reflects a fundamental difference in activation profile, evidenced by the shift in parameter hierarchy across conditions. Under tensile loading, the dominance of offset count (r = 0.807) and segmentation density (r = −0.603) is consistent with interior offset architecture parameters showing stronger and more stable associations under concentrated force application—an interpretation supported by the Kirigami mechanics literature, though not directly instrumented here. Under gravity-drape loading, the relative emergence of boundary curvature effects (C2Radius, nonlinear adj. R^2^ = 0.229) is consistent with large-scale envelope bending showing greater relative statistical weight than interior offset architecture parameters.

As a result, while 44% of displacement variance is shared across regimes (r = 0.665), gravity-drape performance does not constitute a regime-neutral surrogate for tensile performance: the parameter hierarchies shift substantially, and gravity-drape associations are weaker, less Bonferroni-robust, and partly nonlinear. Parameters governing internal architecture—such as offset density and segmentation—are more influential under pull, whereas boundary geometry exerts relatively greater influence under passive loading. This distinction carries a direct design implication: loading regime is not a passive testing condition but an activating context that conditions which geometric parameters govern displacement, rather than a variable placed symmetrically alongside geometry.

Geometric configurations should therefore be evaluated relative to the regime in which they are intended to operate. A pattern optimized for tensile responsiveness may exhibit limited deformation under gravity, and vice versa.

### 7.3. Parameter-Specific Design Implications

The empirical findings can be translated into Parameter-Specific Design Implications that connect geometric parameters to anticipated elastic behavior. These Parameter-Specific Design Implications are not prescriptions; they are bounded propositions derived from observed associations within the tested design space. Their role is to support design-stage reasoning, not to replace validation through fabrication.

Bonferroni correction (α/18 = 0.0028) is used as a confidence filter; parameters not meeting this threshold are interpreted as non-Bonferroni-robust rather than confirmatory, while governance classification remains based on |r| ≥ 0.40, *p* < 0.05.

First, offset count exhibits the strongest cross-regime association with elastic displacement, with Bonferroni-robust evidence under pull and a governing classification under both regimes. If the number of parallel offset curves is increased while overall specimen dimensions are held constant, then pull elastic displacement increases substantially (r = 0.807, 95% CI [0.60, 0.91]; Bonferroni-robust, *p* < 0.001), accounting for approximately 65% of variance (r^2^ = 0.651). A first-order linear approximation derived from a bivariate OLS regression of pull elastic displacement on Number of Offsets (R^2^ = 0.651, F(1, 23) = 42.9, *p* < 0.001) yields: estimated pull displacement (mm) ≈ 424.96 + 16.5 × (Number of Offsets − 32.4). The reference value 424.96 mm is the sample grand mean of pull displacement; 32.4 is the sample mean of Number of Offsets (Table A4). Each additional offset relative to that mean is associated with an estimated difference of approximately 16.5 mm in pull displacement (R^2^ = 0.651); this first-order expression is reported as a screening heuristic and relative-magnitude anchor, not as a predictive model. Under gravity-drape loading, the parameter remains governed by classification criteria (|r| ≥ 0.40, *p* < 0.05), but the reduced magnitude and lack of Bonferroni robustness indicate a weaker and less stable influence relative to pull loading. Implication: increasing offset count is associated with increased elastic displacement across both regimes, with a substantially stronger and more robust effect under tensile loading conditions.

Second, offset distance functions as a pull-exclusive moderator, significant under pull but Non-operative under gravity-drape. If offset distance increases, pull elastic displacement decreases (r = −0.475, *p* = 0.017), while no meaningful association is observed under gravity-drape loading (r = −0.106, *p* = 0.613). The relationship remains stable under shape control but does not meet the Bonferroni correction. Implication: offset spacing is associated as a pull-specific moderator of deformation density; increasing spacing is associated with reduced elastic amplification under tensile loading, while showing no consistent association under passive conditions.

Third, segmentation density governs elastic displacement under pull and modulates it under gravity-drape, with stable directional consistency across regimes. If the number of segments per offset increases, pull elastic displacement decreases (r = −0.603, *p* = 0.001), explaining approximately 36% of variance. Under gravity-drape loading, the association remains directionally consistent but weaker (r = −0.378, *p* = 0.062), classifying the parameter as modulating. Partial correlations confirm stability (r_part = −0.667 pull; −0.384 gravity-drape). Implication: Segmentation density is negatively associated with elastic displacement primarily under tensile loading, with a secondary modulating effect under gravity-drape conditions.

Fourth, outer-boundary void perimeter is the most consistently associated cross-regime parameter, Bonferroni-robust under pull and governing across both regimes. If the length of cut segments along the outer boundary (C2VoidPerimeter) increases, elastic displacement increases under both regimes (pull: r = 0.621, *p* < 0.001; gravity-drape: r = 0.407, *p* = 0.044). The parameter meets the governance threshold under both regimes (|r| ≥ 0.40), with Bonferroni-robust evidence under pull (r = 0.621) and reduced evidential stability under gravity-drape (r = 0.407). Implication: increasing boundary cut length is the most consistently associated cross-regime geometric variable for elastic displacement, with consistent influence independent of shape category.

Fifth, outer curvature exhibits a regime-specific nonlinear association under gravity-drape only. If outer boundary curvature (C2Radiusmm) increases, gravity-drape elastic displacement increases through a nonlinear relationship (cubic model adj. R^2^ = 0.229, *p* = 0.022). No significant association is observed under pull loading, and the effect does not meet the Bonferroni correction. Implication: outer curvature operates as a regime-specific, nonlinear governing influence under gravity-drape conditions; its application should remain bounded by the tested parameter range given the absence of Bonferroni robustness.

The five Parameter-Specific Design Implications represent conditional relationships between geometric parameters and elastic response; however, their design value depends on how they are applied in combination rather than interpreted in isolation. These individual parameter effects do not operate independently, but are contingent on loading regime, parameter coupling, and performance intent. As such, the Parameter-Specific Design Implications alone do not yet constitute a usable design procedure. Designers require a structured means of coordinating these conditional propositions, determining parameter priority, and iteratively aligning geometric configuration with expected behavior. The following section addresses this need by organizing the Parameter-Specific Design Implications into an application structure that supports design-stage configuration within the tested design space.

### 7.4. Bio-Inspired Regime-Dependent Parameter Selection in Parametric Kirigami

**Figure 8:**
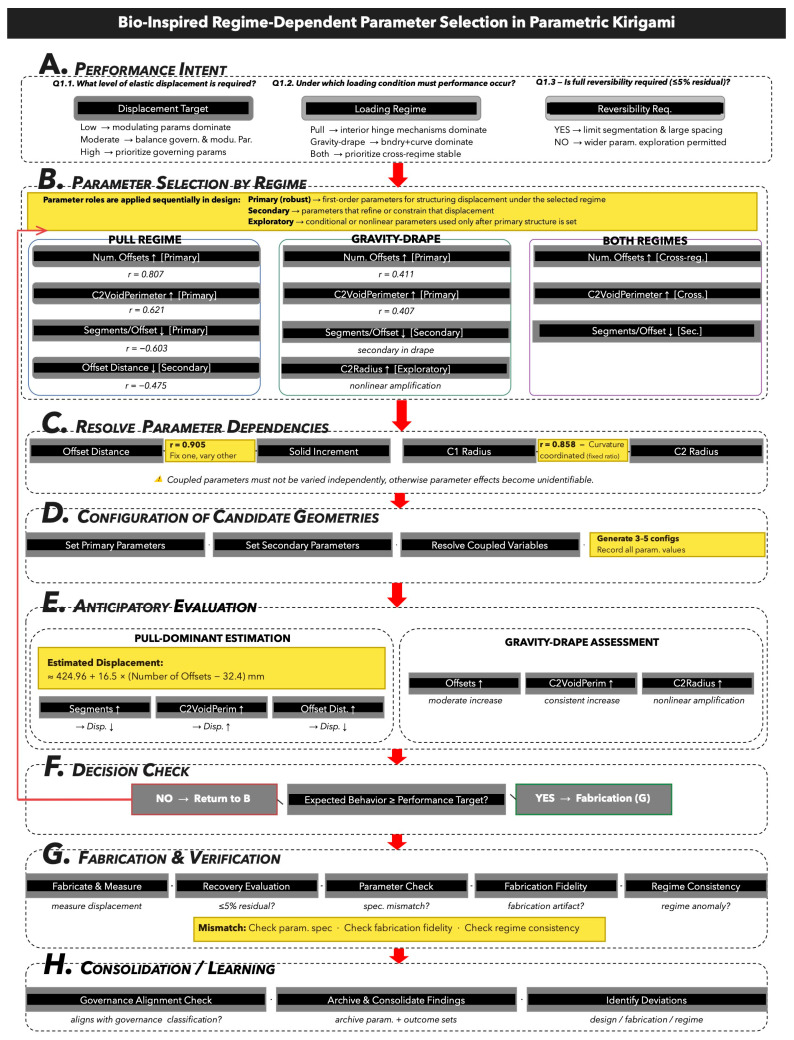
Bio-inspired regime-dependent parameter selection in parametric Kirigami.

The Bio-Inspired Regime-Dependent Parameter Selection in Parametric Kirigami, as presented in Figure 8, structures the application of Parameter-Specific Design Implications to configure Kirigami geometry prior to fabrication. Its purpose is not to predict displacement precisely, but to structure parameter interaction, support identification of potentially contradictory configurations, and enable target-oriented selection of feasible geometries within the tested design space.

The Kirigami design starts by fixing performance intent (A), which establishes the evaluation benchmark. For example, a target displacement of 300 mm under pull loading with ≤5% residual deformation defines the acceptable range, the intended loading regime, and the admissible geometric behavior. This immediately activates a parameter hierarchy (B), summarized in Table 14: offset count and boundary void perimeter operate as primary drivers, while segmentation and spacing act as constraints on that capacity. Without this ordering, parameter variation becomes non-directional, and effects cannot be attributed.

Parameter dependencies (C) are resolved before any geometry is generated. The strong couplings identified in Table 14—Offset Distance–Solid Increment (r = 0.905) and C1–C2 Radius (r = 0.858)—are treated as single degrees of freedom: one variable is fixed, or both are coordinated (e.g., a constant curvature ratio), ensuring that subsequent changes in displacement can be attributed to identifiable parameters rather than conflated effects.

Candidate geometries (D) are then constructed as controlled variations, not open exploration. A small set (e.g., 3–5 configurations) is generated by varying primary parameters while holding dependent and secondary parameters within constrained ranges. For instance: Config A: Offsets = 12, Segments = high, C2Void = moderate; Config B: Offsets = 18, Segments = low, C2Void = high; Config C: Offsets = 24, Segments = low, C2Void = high.

**Table 14:** Application role of parameters for the pull and gravity-drape regimes.

Parameter	Pull Regime	Gravity-Drape Regime
Number of Offsets	Primary (r = 0.807, Bonferroni)	Primary (r = 0.411)
Offset Distance	Secondary (Constraint) (r = −0.475; coupled with Solid Increment, r = 0.905)	Exploratory (Inactive) (r = −0.106; coupled with Solid Increment)
Segments per Offset	Primary (Constraint) (r = −0.603, Bonferroni)	Secondary (Modulating) (r = −0.378)
C2 Void Perimeter	Primary (r = 0.621, Bonferroni)	Primary (r = 0.407)
C2 Perimeter	Secondary (Modulating) (r = 0.418 †)	Secondary (Modulating) (r = 0.302)
C2 Radius	Exploratory (Weak) (r = 0.377; coupled with C1 Radius, r = 0.858)	Governing (Nonlinear, bounded) (adj. R^2^ = 0.229; coupled with C1 Radius)
C1 Radius	Exploratory (Inactive) (r = 0.131; coupled with C2 Radius, r = 0.858)	Exploratory (Inactive) (r = 0.392 †; coupled with C2 Radius)
Solid Increment	Secondary (Dependent constraint) (r = −0.356; coupled with Offset Distance, r = 0.905)	Secondary (Dependent constraint) (r = 0.010; coupled with Offset Distance)
Solid Start Length	Exploratory (Inactive) (r = −0.023)	Exploratory (Inactive) (r = 0.176)

† Indicates attenuation under shape-controlled partial correlation (see Table 13), resulting in reclassification where applicable.

Evaluation (E) functions as a pre-fabrication screening step. Under pull loading, a first-order estimate is obtained from offset count: A → 424.96 + 16.5 × (12 − 32.4) ≈ 88 mm; B → 424.96 + 16.5 × (18 − 32.4) ≈ 187 mm; C → 424.96 + 16.5 × (24 − 32.4) ≈ 286 mm. This estimate establishes relative magnitude only. It is then interpreted per configuration through directional adjustments based on parameter composition: if segmentation is high → the estimated displacement for that configuration is expected to decrease relative to its offset-based value; if C2VoidPerimeter is high → the estimated displacement for that configuration is expected to increase; if offset distance is large → hinge activation is reduced, lowering the effective displacement for that configuration.

These adjustments do not modify the numerical estimate directly; they provide qualitative correction cues that refine how each configuration is interpreted relative to the performance target. The outcome is not a final prediction, but a ranking of configurations relative to the 300 mm target.

Under a gravity-drape, no numerical estimate is applied. Instead, configurations are assessed directionally (e.g., offsets ↑ → moderate increase; C2Void ↑ → consistent increase; curvature ↑ → nonlinear amplification), preventing false precision where no robust predictive relationship exists.

The decision condition (F) accepts or rejects configurations based on whether expected behavior aligns with the defined target. Rejected configurations are not fabricated; parameter selection is revised, typically by adjusting governing variables or resolving constraint violations. This establishes a closed-loop at the parameter level, avoiding iterative fabrication cycles.

Fabrication and verification (G) test the selected configuration. Deviations are interpreted diagnostically: whether caused by parameter mis-specification, fabrication artifacts, or regime inconsistency. This maintains separation between design logic and production error.

Finally, consolidation (H) records parameter–performance relationships and checks alignment with governance classification, enabling accumulation of structured design knowledge across iterations.

In this parameter selection procedure, the offset-based estimate provides a magnitude anchor, but the design outcome is determined by how parameters are prioritized, constrained, and combined. The parameter selection procedure is therefore necessary not to increase predictive accuracy, but to support consistency in geometric configuration relative to the intended performance condition, reducing the risk of internally contradictory parameter assignments prior to fabrication.

### 7.5. Worked Example: Selecting Parameters for a Bio-Inspired Adaptive Facade Panel

To illustrate how the parameter selection procedure operates within the tested design space, consider a hypothetical adaptive-facade panel inspired by the deployment logic of insect hindwings [29,30]: a 267 mm × 267 mm thin-sheet element required to undergo approximately 600 mm of reversible elastic displacement under an applied tensile cue (e.g., a cable retraction mechanism) and to drape passively under self-weight when the cue is released. The intended performance is therefore tensile-dominant, with a secondary gravity-drape requirement. Step 1 (regime selection) identifies pull as the primary activation regime, since the controlled actuation event is tensile. Step 2 (parameter hierarchy) selects from the parameters identified as governing under pull: Number of Offsets (r = 0.807, Bonferroni-robust), Segments per Offset (r = −0.603, Bonferroni-robust), and outer-boundary void perimeter (r = 0.621, Bonferroni-robust). Step 3 (magnitude anchor) applies the first-order linear estimate from Section 7.3: required pull displacement of 600 mm corresponds to approximately 32 + (600 − 424.96)/16.5 ≈ 43 offsets within the tested design space. Step 4 (modulation) adjusts Segments per Offset downward from the sample mean to amplify compliance, and adjusts boundary cut perimeter upward, both consistent with their directional associations. Step 5 (regime-pure secondary check) verifies that the resulting configuration does not place outer curvature in a range that would couple strongly under gravity-drape, since C2 Radius is regime-sensitive under passive loading. Step 6 (consolidation) records the proposed configuration for prospective fabrication and prospective validation. This vignette is illustrative rather than validated: it applies the selection procedure as a semi-formal decision heuristic under bounded empirical support, demonstrating how the screening-level associations translate into a pre-fabrication parameter set. The 600 mm target lies above the sample mean (425 mm) but within the observed range of pull displacement (40–950 mm), and the implied configuration (≈43 offsets) lies within the tested range (15–52 offsets); the example is therefore an in-range but unvalidated illustration that would require confirmatory fabrication to verify behavior in the recommended configuration.

## 8. Discussion

The present study establishes empirical and methodological groundwork for approaching Kirigami elastic displacement as a bio-inspired, regime-dependent parameter-selection problem within a bounded parametric design space. In much of the existing literature, elastic behavior is demonstrated through fabricated examples and then interpreted through geometry or mechanics after testing [4,6,10]. By distinguishing Governing, Modulating, and Non-operative parameters, the study provides a structured basis for deciding which geometric variables merit priority before fabrication and which have limited behavioral leverage within the tested conditions. Within this design space, geometry is positioned not only as a field of formal variation but as a field of unequal deformation capacity: a small subset of parameters accounts for the dominant share of variation in elastic displacement, while others modulate or leave displacement weakly affected. The practical implication, if this structure is confirmed across larger and more varied samples, is that bio-inspired Kirigami configuration need not proceed through simultaneous variation in all parameters. Beginning from the parameters with the strongest and most stable governance—offset count and boundary void perimeter—and using secondary variables to refine rather than define behavior represents a more directed selection logic. Whether this reduces undirected iteration in practice remains an empirical question; the present study establishes the parameter structure that would make such an approach analytically coherent, but prospective validation of its efficiency against standard iterative methods is required before this claim can be made with confidence.

The importance of regime-dependence is equally substantive. A central implication of the findings is that elastic behavior cannot be treated as intrinsic to geometric configuration alone. The same specimen does not express the same deformation logic under pull and gravity-drape loading, with different force conditions corresponding to systematically different parameter-level associations. Under tensile loading, internal offset architecture parameters show stronger and more Bonferroni-robust associations with displacement; under gravity-drape, boundary and envelope effects become relatively more consequential. It alters how Kirigami should be specified in bio-inspired design contexts. In design terms, the loading regime becomes part of the parameter-selection problem itself, not a post hoc testing condition. One implication that falls outside the present scope but warrants explicit acknowledgment is the case in which both regimes are simultaneously relevant—for instance, in applications requiring tensile responsiveness and stable gravity-drape behavior within the same configuration. Given that governing parameters shift substantially across regimes, such dual-requirement cases may involve parameter conflicts not resolvable through the single-regime priority logic of the present procedure. This constitutes a direct motivation for multi-regime optimization as a subsequent design problem. This aligns with broader arguments in materials design that behavior emerges from the interaction between structure, processing conditions, and use rather than from form alone.

The study also suggests a more precise understanding of what parametric control should mean in bio-inspired Kirigami design. Parametric modeling often implies open-ended variation; here, the findings argue for selective variation. Strong coupling between certain variables, such as offset distance with solid increment and C1 with C2 radius, shows that not all parameter changes are interpretable as independent design moves. When coupled variables are varied indiscriminately, apparent behavioral effects become difficult to attribute, and the design space becomes formally rich but analytically opaque. The parameter selection procedure counters this by linking parameter variation to interpretability. In that sense, it does not merely identify effective variables; it clarifies the conditions under which geometric variation remains meaningful for elastic displacement.

At the same time, the scope of the present contribution must remain explicit. The study is exploratory, based on a limited sample (*N* = 25) within fixed dimensional and material ranges. Only three pull-regime associations meet the Bonferroni-corrected threshold, and the gravity-drape relationships remain weaker, more conditional, and in part nonlinear. The parameter selection procedure should therefore be understood as a bounded design instrument rather than a universal rule set. Its value lies in establishing a credible structure for design-stage reasoning within the tested design space and in defining a methodological basis for confirmatory extension. Future work should test whether the same governance hierarchy persists across larger factorial samples, instrumented force–displacement protocols, and broader material systems. Even within these limits, the study establishes one design-relevant distinction: in bio-inspired Kirigami design, the critical question is no longer only what geometry resembles or produces formally, but which parts of geometry govern what the system can do under a specified loading regime.

## 9. Limitations and Future Work

The findings are derived from an exploratory dataset (*N* = 25) within a bounded parametric design space and must be interpreted within this scope. Statistical power is limited for detecting moderate associations (|r| ≈ 0.40), introducing the possibility that parameters classified as Non-operative or modulating may reflect under-detection rather than absence of effect. The three Bonferroni-robust relationships under pull loading—offset count (r = 0.807), segmentation density (r = −0.603), and boundary void perimeter (r = 0.621)—exceed the minimum detectable effect size and therefore define the most reliable empirical structure in the dataset. All remaining associations, particularly under gravity-drape loading, should be treated as provisional classifications pending confirmation in larger samples. In addition, geometric ranges, including curvature, spacing, segmentation, and boundary typologies, are constrained; parameter roles established here are therefore valid within the tested design space and may shift under expanded geometric or boundary conditions.

Elastic displacement is defined as recoverable geometric deformation rather than constitutive material elasticity. The absence of instrumented force–displacement measurement limits interpretation to kinematic response and precludes direct characterization of stiffness, energy absorption, or material modulus. Manual pull loading, while consistently applied, does not provide calibrated force control. The protocol does not address cyclic loading, rate dependence, or long-term material response, which are critical for repeated or adaptive applications. Material effects are interpreted conservatively: substrates were chemically identified by FTIR, DSC, and XRD (Section 4.1) and differ in thickness and surface condition, but their calibrated mechanical constants (modulus, yield, viscoelastic parameters) were not measured, which is what limits the separation of geometric and material contributions. Strong parametric coupling within the generative logic further constrains independent variation, limiting the extent to which parameter effects can be isolated under simultaneous change.

Within these constraints, the study suggests preliminary evidence of a conditionally associative structure with limited predictive implications between geometric configuration and elastic displacement. Under tensile loading, displacement can be quantitatively estimated from offset architecture, for example, 424.96 + 16.5 × (# of Offsets − 32.4), with systematic adjustment through segmentation and boundary void parameters. Under gravity-drape loading, prediction remains regime-specific and directional, reflecting weaker, partly nonlinear associations that do not meet the Bonferroni correction. Parameter governance may support the specification of deformation outcomes prior to fabrication by prioritizing governing variables, resolving dependencies, and aligning configuration with loading regime; prospective validation is required to confirm this capacity. The parameter selection procedure is intended to reduce reliance on unconstrained iteration by replacing non-directional trial with parameter-directed configuration within a defined design space; this benefit has not yet been empirically tested.

The experimental protocol applies pull loading before gravity-drape loading across all specimens. While this sequence was held constant, residual deformation from pull testing—even within the ≤5% recovery threshold—may have introduced systematic baseline shifts in panel planarity prior to gravity-drape measurement. This ordering effect cannot be disentangled from regime-specific geometric associations in the present dataset, and represents a limitation of the single-sequence protocol. Counterbalanced or between-subjects loading designs would be required to isolate regime-pure behavioral profiles.

Future work proceeds along two coupled directions: confirmatory validation and application expansion. Confirmatory studies should employ larger samples (*N* ≥ 60–80), factorial variation in primary parameters, and instrumented force–displacement measurement to validate governance thresholds and quantify mechanical response. Expanding geometric ranges is essential to test whether parameters classified here as Non-operative or modulating become governing under alternative configurations. Additional material systems with characterized properties, variation in boundary conditions, and testing under cyclic and multi-regime loading will establish the stability of the parameter selection procedure beyond the present experimental conditions.

In parallel, the parameter selection procedure should be extended toward performance-oriented applications in adaptive architecture and product design. In architectural systems, this includes responsive facades, deployable shading elements, and interior assemblies capable of controlled geometric reconfiguration under environmental or user-induced loading. In product design, the same parameter-governed logic can inform compliant mechanisms, transformable surfaces, and material-efficient structures where deformation is intentionally programmed [44]. In these contexts, the parameter selection procedure may support specification of displacement magnitude, reversibility thresholds, and activation conditions at the design stage, constraining exploration to configurations with better-defined behavioral tendencies and thereby reducing reliance on material-intensive iteration. Among these, a directly biomimetic architectural application is the water-shedding building envelope: just as the coupling geometry of beetle elytra unites deployability with water-proofing [31], regime-tuned Kirigami skins could couple controlled geometric reconfiguration with passive water-shedding across roofs, façades, and deployable canopies. Comparable structure–function integration extends beyond architecture to deployable and soft-robotic surfaces, where a single cut geometry is required to govern more than one performance criterion at once [31,44].

Taken together, these directions position the parameter selection procedure as both an applied design instrument and a foundation for establishing more generalizable geometric–behavior mappings. Its current contribution is bounded but operational: it demonstrates that elastic displacement in parametric Kirigami can be approached through bio-inspired, regime-dependent geometric configuration, within the empirical scope of the tested design space.

## 10. Conclusions

Elastic displacement in Kirigami systems cannot be attributed to geometric configuration in isolation. The results indicate that deformation behavior is structured through a conditional relationship in which geometric parameters operate at different levels of governance and are subject to regime-contingent associations with elastic displacement. Offset architecture, segmentation density, and boundary void perimeter are the primary geometric variables associated with elastic displacement, but their influence is not invariant; it is contingent on how force is introduced and distributed. Geometry, in this sense, supports potential rather than prescribes outcome.

This shifts the locus of design from form generation to regime-aligned parameter selection. Within the tested design space, elastic performance need not be discovered solely after fabrication; it may be approached through the coordination of parameter hierarchy, dependency resolution, and loading context. The parameter selection procedure developed in this study supports this shift by linking performance intent to geometric configuration through condition-dependent reasoning, supporting design-stage estimation and configuration of displacement within defined scope conditions.

The implication is direct: behavior is not contained within geometry—it emerges through activation. These findings support the broader biomimetic proposition that adaptive thin-sheet behavior emerges not from intrinsic material properties but from the spatial organization of compliance, segmentation, and curvature—and that the parameters governing this organization are themselves regime-dependent in engineered Kirigami as they are in their biological exemplars (leaves, insect wings, flowering organs).

## Figures and Tables

**Table 2:** Governance classification stability across analytic thresholds (11 parameters × 4 analytic thresholds; adopted threshold |r| ≥ 0.40; *N* = 25).

Parameter	Loading Regime	|r|	|r| ≥ 0.30	|r| ≥ 0.35	|r| ≥ 0.40 (Adopted)	|r| ≥ 0.45
★ No. of Offsets	Pull	0.807	Governing	Governing	Governing	Governing
★ C2 Void Perimeter	Pull	0.621	Governing	Governing	Governing	Governing
★ Segments/Offset	Pull	−0.603	Governing	Governing	Governing	Governing
Offset Distance	Pull	−0.475	Governing	Governing	Governing	Below threshold
No. of Offsets	Gravity-drape	0.411	Governing	Governing	Governing	Below threshold
C2 Void Perimeter	Gravity-drape	0.407	Governing	Governing	Governing	Below threshold
C2 Perimeter	Pull	0.418	Governing	Governing	Modulating †	Below threshold
Segments/Offset	Gravity-drape	0.378	Governing	Modulating	Modulating	Below threshold
Solid Increment	Pull	−0.356	Governing	Modulating	Modulating	Non-operative
C2 Radius	Gravity-drape	NL	Below threshold	Below threshold	Below threshold	Below threshold
C1 Radius	Pull	0.131	Non-operative	Non-operative	Non-operative	Non-operative

★ Bonferroni-robust (*p* < 0.003). NL = nonlinear model. Note. Adopted threshold: |r| ≥ 0.40. Three Bonferroni-robust associations significant at corrected α = 0.0028 (18 simultaneous tests): No. of Offsets Pull, C2 Void Perimeter Pull, Segments/Offset Pull—Governing across all four thresholds. C2 Radius Gravity-drape classified via nonlinear regression (Table 6), not Pearson r. C2 Perimeter Pull classified as Modulating † despite bivariate r = 0.418 > 0.40; partial correlation attenuates to r_part = 0.371 (Table 13).

**Table 3:** Summary correlation matrix.

Parameter	Pull r	Pull p	Gravity-Drape r	Gravity-Drape p	Pull Class.	Gravity-Drape Class.
★ Number of Offsets	0.807	<0.001 ✦	0.411	0.041	Governing ★	Governing
Offset Distance	−0.475	0.017	−0.106	0.613	Governing	Non-operative
★ Segments per Offset	−0.603	0.001 ✦	−0.378	0.062	Governing ★	Modulating
★ C2 Void Perimeter	0.621	<0.001 ✦	0.407	0.044	Governing ★	Governing
C2 Perimeter	0.418	0.038	0.302	0.142	Modulating †	Modulating
C2 Radius	0.377	0.064	0.414	0.040	Modulating	Governing (NL)
C1 Radius	0.131	0.533	0.392	0.052	Non-operative	Modulating
Solid Increment	−0.356	0.081	0.010	0.963	Modulating	Non-operative
Solid Start Length	−0.023	0.914	0.176	0.401	Non-operative	Non-operative
Pull–Gravity-drape Correlation	0.665	<0.001 ✦	—	—	—	—

★ Bonferroni-robust. ✦ *p* < 0.0028. † Partial r after shape control falls below threshold (Table 13). (NL) Classified via nonlinear regression (Table 6). Note. Pearson r values with two-tailed significance. Bonferroni-robust threshold: α/18 = 0.0028. Governance: governing (|r| ≥ 0.40, *p* < 0.05); Modulating (|r| ≥ 0.30); Non-operative (|r| < 0.30). *n* = 25.

**Table 6:** Nonlinear regression models—C2 Radius (outer curvature). Elastic displacement as outcome variable. Pull and Gravity-drape regimes. *N* = 25.

Model	Pull R^2^	Pull Adj. R^2^	Pull SE	Pull F (df)	Pull p	GD R^2^	GD Adj. R^2^	GD SE	GD F (df)	GD p
Quadratic f(C2, C2^2^)	0.159	0.083	229.86	2.082 (2, 22)	0.149	0.287	0.222	147.09	4.432 (2, 22)	0.024
Cubic f(C2, C2^3^ ^a^)	0.160	0.084	229.76	2.094 (2, 22)	0.147	0.294	0.229	146.43	4.573 (2, 22)	0.022
Piecewise k = 90 mm	0.142	0.104	227.13	3.799 (1, 23)	0.064	0.171	0.135	155.14	4.744 (1, 23)	0.040

Note. OLS regression; two-tailed *p*-values. *N* = 25. Pull and Gravity-drape elastic displacement as outcome variables. C2 = outer curvature radius. ^a^ Cubic model estimated as C2_C + C2_C^3^; squared term omitted due to near-collinearity. Gravity-drape regime models reach significance (*p* < 0.05) for Quadratic and Cubic; the Piecewise model is marginal (*p* = 0.040). Pull regime remains non-significant across all models (*p* > 0.06). GD = Gravity-drape.

**Table 7:** Shape category Pearson correlations with elastic displacement under pull and gravity-drape loading (*N* = 25, two-tailed).

Shape Category	Pull r	Pull p	Gravity-Drape r	Gravity-Drape p	Assessment
D_Circle (*n* = 9)	0.065	0.759	0.304	0.139	Non-operative (both regimes)
D_Square (*n* = 5)	0.150	0.475	−0.014	0.947	Non-operative (both regimes)
D_Triangle (*n* = 9)	−0.243	0.243	−0.261	0.207	Non-operative; consistent negative direction
D_Pentagon (*n* = 1; descriptive only)	0.082	0.695	−0.001	0.997	Uninterpretable (*n* = 1)
D_Hexagon (*n* = 1; descriptive only)	0.048	0.821	−0.076	0.719	Uninterpretable (*n* = 1)

**Table 8:** Two-way general linear model (Type III): material type and loading mode effects on Kirigami elasticity (ICC ≈ 0). *N* = 50 observations (25 specimens). R^2^ = 0.859 (adjusted R^2^ = 0.808).

Source	df	F	*p*	Partial η^2^	Model Role
Material	2	4.254	0.022	0.189	Significant main effect
Loading Mode	1	109.568	<0.001	0.766	Dominant main effect
Material × Mode	2	2.391	0.106	0.125	Moderate, non-significant
Offset Distance (mm)	1	<0.001	0.996	0.000	Non-significant covariate
Number of Offsets	1	3.004	0.092	0.077	Non-significant covariate
Number of Segments per Offset	1	32.535	<0.001	0.475	Significant covariate †
Solid Start Length (mm)	1	0.191	0.664	0.005	Non-significant covariate
Solid Increment (mm)	1	0.038	0.847	0.001	Non-significant covariate
C1 Radius (mm)	1	8.479	0.006	0.191	Significant covariate †
C2 Radius (mm)	1	3.379	0.074	0.086	Non-significant covariate
C2 Perimeter	1	10.217	0.003	0.221	Significant covariate †

Note. Model fit: R^2^ = 0.859 (adjusted R^2^ = 0.808); ICC ≈ 0. Reported F-values, degrees of freedom, and partial η^2^ (Type III sums of squares). † Statistically significant covariate (*p* < 0.05); significance does not determine governance classification.

## Data Availability

The dataset—geometric parameter measurements, elastic displacement values, and specimen category labels for *N* = 25 specimens—is available from the corresponding author upon reasonable request. The full reproducibility package for parametric Grasshopper models (Rhinoceros 3D) that generate all specimen geometries is also available upon request.

## Abbreviations

The following abbreviations are used in this manuscript:

**Abbreviation**	**Meaning**
BR	Bonferroni-Robust
NBR	Non-Bonferroni-Robust
BM	Boundary Metric
CC	Categorical Covariate
DV	Dependent Variable
GLM	General Linear Model
IV	Independent Geometric Variable
ICC	Intraclass Correlation Coefficient
NL	Nonlinear
OLS	Ordinary Least Squares
REML	Restricted Maximum Likelihood
SD	Standard Deviation
SE	Standard Error
SEE	Standard Error of the Estimate
VIF	Variance Inflation Factor

## Appendix A

### Appendix A.1. Collinearity Diagnostics—Full OLS Regression Output

OLS regression of pull elastic displacement (mm) on all eight geometric covariates (*N* = 25). The model confirms severe multicollinearity across the predictor set: five of eight predictors exhibit VIF > 10. The full OLS coefficients are reported for transparency and to support the VIF interpretation in Section 5.4; regression coefficients should not be interpreted as independent effects given the collinearity context. Dependent variable: pull elastic displacement (mm). The model is estimated solely to diagnose collinearity, not to infer independent parameter effects.

**Table A1:** Model summary.

R	R^2^	Adjusted R^2^	SEE	F	df_1_, df_2_	*p*
0.968	0.937	0.906	73.509	29.980	8, 16	<0.001

**Table A2:** Coefficients and collinearity statistics.

Parameter	B	SE	β	t	Sig.	Tolerance	VIF
(Constant)	−7.027	196.410	—	−0.036	0.972	—	—
Offset Distance mm	−25.362	58.653	−0.136	−0.432	0.671	0.039	25.466
Number of Offsets	8.634	6.031	0.423	1.432	0.171	0.045	22.336
Number of Segments per Offset	−48.249	6.282	−0.544	−7.681	<0.001	0.778	1.285
Solid Start Length mm	−16.341	26.980	−0.063	−0.606	0.553	0.365	2.736
Solid Increment mm	−62.930	148.885	−0.085	−0.423	0.678	0.096	10.410
C1 Radius mm	10.235	9.475	0.260	1.080	0.296	0.067	14.824
C2 Radius mm	−1.498	3.544	−0.149	−0.423	0.678	0.031	31.805
C2 Perimeter	1.137	0.357	0.462	3.186	0.006	0.186	5.387

Note. Rows with VIF > 10 indicate severe multicollinearity. Dependent variable: pull elastic displacement (mm). *N* = 25; df_1_ = 8, df_2_ = 16. SEE = standard error of the estimate. Tolerance = 1/VIF. Condition index for the final eigenvalue dimension = 85.859.

### Appendix A.2. Pearson Correlations Matrix

This table is developed using SPSS version 31.0.2.0(126). All pairs with |r| > 0.80 are excluded from simultaneous regression models. Significance levels: * *p* < 0.05; ** *p* < 0.01; *** *p* < 0.001 (two-tailed).

**Table A3:** Pearson correlation matrix between elastic displacement outcomes and geometric parameters (*N* = 25).

	GD	Pull	OD	NO	NSO	SSL	SI	C1R	C2R	C2P	C2VP
Eval_Elasticity (Gravity-drape) mm (GD)	1	0.665 ***	−0.106	0.411 *	−0.378	0.176	0.010	0.392	0.414 *	0.302	0.407 *
Eval_Elasticity (Pull) mm	0.665 ***	1	−0.475 *	0.807 ***	−0.603 **	−0.023	−0.356	0.131	0.377	0.418 *	0.621 ***
Offset Distance mm (OD)	−0.106	−0.475 *	1	−0.708 ***	0.191	0.292	0.905 ***	0.524 **	0.388	0.178	0.047
Number of Offsets (NO)	0.411 *	0.807 ***	−0.708 ***	1	−0.221	−0.186	−0.537 **	0.041	0.333	0.322	0.342
Number of Segments per Offset (NSO)	−0.378	−0.603 **	0.191	−0.221	1	0.131	0.257	0.049	0.034	0.181	−0.215
Solid Start Length mm (SSL)	0.176	−0.023	0.292	−0.186	0.131	1	0.427 *	0.365	0.195	0.433 *	0.432 *
Solid Increment mm (SI)	0.010	−0.356	0.905 ***	−0.537 **	0.257	0.427 *	1	0.679 ***	0.528 **	0.321	0.111
C1 Radius mm (C1R)	0.392	0.131	0.524 **	0.041	0.049	0.365	0.679 ***	1	0.858 ***	0.347	0.164
C2 Radius mm (C2R)	0.414 *	0.377	0.388	0.333	0.034	0.195	0.528 **	0.858 ***	1	0.628 ***	0.434 *
C2 Perimeter (C2P)	0.302	0.418 *	0.178	0.322	0.181	0.433 *	0.321	0.347	0.628 ***	1	0.889 ***
C2 Void Perimeter (C2VP)	0.407 *	0.621 ***	0.047	0.342	−0.215	0.432 *	0.111	0.164	0.434 *	0.889 ***	1

*N* = 25 for all variables. Significance: * *p* < 0.05; ** *p* < 0.01; *** *p* < 0.001 (two-tailed). Highly positive coupling: Offset Distance ↔ Solid Increment (r = 0.905). Highly positive couplings include C1 ↔ C2 Radius (r = 0.858) and C2 Perimeter ↔ C2 Void Perimeter (r = 0.889).

### Appendix A.3. Descriptive Statistics of Independent and Dependent Variables

Descriptive statistics for all geometric independent variables and both dependent variables (*N* = 25 specimens), derived from SPSS Descriptives output (version 31.0.2.0(126)). Values reflect the final analysis sample following exclusions described in Section 4.4.

**Table A4:** Descriptive statistics for geometric parameters and elastic displacement outcomes (*N* = 25).

Variable	*N*	Range	Min	Max	Mean	SD
Offset Distance, mm	25	4	2	6	2.80	1.291
Number of Offsets	25	37	15	52	32.40	11.758
Number of Segments per Offset	25	10	6	16	9.60	2.708
Solid Start Length, mm	25	2.4	2.0	4.4	3.584	0.920
Solid Increment, mm	25	1.2	0.0	1.2	0.464	0.325
C1 Radius, mm	25	15.0	5.0	20.0	11.548	6.097
C2 Radius, mm	25	55.0	63.0	118.0	89.548	23.880
C2 Perimeter	25	493.3	427.3	920.6	694.872	97.591
C2 Void Perimeter	25	431.4	341.0	772.4	586.685	85.755
Eval_Elasticity (Pull), mm	25	910	40	950	424.96	240.005
Eval_Elasticity (Gravity-drape), mm	25	805.5	−8.0	797.5	68.220	166.805

*N* = 25 for all variables. SD = standard deviation.

### Appendix A.4. Sensitivity Analysis—C2VoidPerimeter Specification

Supplementary fixed-effects general linear model (Type III) with C2VoidPerimeter as the primary boundary covariate. C2Perimeter is excluded due to near-perfect multicollinearity with C2VoidPerimeter (r = 0.889, *p* < 0.001). All other model specifications match the primary model reported in Section 5.4.1. The material main effect remains statistically significant (F(2, 36) = 4.098, *p* = 0.025), confirming robustness to boundary parameter specification. Specimen-level variance was negligible (σ^2^_specimen ≈ 0; ICC ≈ 0).

**Table A5:** Sensitivity model results (fixed-effects GLM): Type III fixed effects with C2VoidPerimeter as boundary covariate (*N* = 50 observations, 25 specimens; df_residual = 36).

Source	df	F	*p*	Partial η^2^
Material (Material_num)	2	4.098	0.025	0.185
Loading Mode (Pull vs. Gravity-drape)	1	112.237	<0.001	0.757
Material × Mode interaction	2	2.449	0.101	0.120
OffsetDistancemm	1	0.209	0.651	0.006
NumberofOffsets	1	2.617	0.114	0.068
NumberofSegmentsperOffset	1	14.879	<0.001	0.292
SolidStartLengthmm	1	0.368	0.548	0.010
SolidIncrementmm	1	0.790	0.380	0.021
C1Radiusmm	1	8.622	0.006	0.193
C2Radiusmm	1	2.443	0.127	0.063
C2VoidPerimeter	1	11.343	0.002	0.240

Model fit: R^2^ = 0.862 (adjusted R^2^ = 0.812); ICC ≈ 0. Partial η^2^ = (F × df_num)/(F × df_num + df_denom); df_denom = 36.

### Appendix A.5. Primary Model Variance Components and Fit (Fixed-Effects GLM; C2Perimeter)

The primary model uses C2Perimeter as the boundary covariate (cf. Appendix A.4, which uses C2VoidPerimeter); all other specifications match Section 5.4.1 and Table 8. *N* = 50 observations, 25 specimens; df_residual = 36 (SPSS, version 31.0.2.0(126)). Specimen-level variance was negligible (σ^2^_specimen ≈ 0; ICC ≈ 0), so material type and loading mode were modeled with the geometric covariates as fixed effects. The variance components below report the results.

**Table A6:** Primary model variance components and fit (fixed-effects general linear model; C2Perimeter as boundary covariate).

Parameter	Value
Residual variance (σ^2^_residual)	14,300.684
Specimen intercept variance (σ^2^_specimen)	0.000
Intraclass Correlation Coefficient (ICC)	≈0
−2 Restricted Log Likelihood	501.687
AIC	505.687
BIC	508.855
R^2^ (fixed-effects GLM)	0.859
Adjusted R^2^	0.808

This covariance parameter is redundant; σ^2^_specimen = 0.000 confirms ICC ≈ 0. With σ^2^_specimen = 0.000, a specimen random term would contribute no explained variance beyond the fixed effects; the model is therefore specified with fixed effects only (R^2^ = 0.859; adjusted R^2^ = 0.808). Fixed-effect Type III results are reported in Table 8 (Section 5.4.1). For the sensitivity model, substituting C2VoidPerimeter for C2Perimeter, see Appendix A.4.

### Appendix A.6. Principal-Component Analysis of the Coupled Predictor Block

To characterize the dimensionality of the coupled geometric predictors, a principal-component analysis was conducted on the eight standardized predictors entered in the collinearity diagnostics (Appendix A.1; *N* = 25). The analysis decomposes the correlation structure of the predictor block and is reported to document the latent geometric dimensionality underlying the parametric construction; it does not enter the association analyses and does not alter any reported correlation, governance classification, or model estimate. Three components have eigenvalues above 1.0 and together account for 82.3% of the total predictor variance, with the first two components accounting for 69.1% (Table A7). The strongly coupled parameters load together on the first component (Table A8): Solid Increment (−0.92), Offset Distance (−0.82), C1 Radius (−0.82), and C2 Radius (−0.74) share the dominant axis, consistent with their high pairwise correlations (Offset Distance–Solid Increment, r = 0.905; C1–C2 Radius, r = 0.858). Therefore, the analysis confirms that the nominally distinct geometric variables collapse onto a small number of latent geometric axes, providing the empirical basis for treating strongly coupled pairs as single design degrees of freedom (Section 4.6).

**Table A7:** Eigenvalues and variance explained by the principal components of the eight-predictor geometric block (standardized; *N* = 25).

Component	Eigenvalue	% Variance	Cumulative %
PC1	3.535	44.2	44.2
PC2	1.994	24.9	69.1
PC3	1.052	13.1	82.3
PC4	0.828	10.4	92.6
PC5	0.461	5.8	98.4
PC6	0.074	0.9	99.3
PC7	0.043	0.5	99.8
PC8	0.014	0.2	100.0

**Table A8:** Component loadings (component–variable correlations) for the first three principal components (eigenvalues > 1.0).

Parameter	PC1	PC2	PC3
Offset Distance	−0.82	0.47	−0.19
Number of Offsets	0.31	−0.93	0.03
Segments per Offset	−0.28	0.23	0.73
Solid Start Length	−0.56	−0.01	0.41
Solid Increment	−0.92	0.28	−0.10
C1 Radius	−0.82	−0.30	−0.30
C2 Radius	−0.74	−0.59	−0.21
C2 Perimeter	−0.55	−0.57	0.41

Note. Principal-component analysis of the standardized eight-predictor geometric block (Appendix A.1); extraction by eigen-decomposition of the correlation matrix. Loadings are component–variable correlations. The analysis is descriptive of the predictor structure and does not affect any association, governance, or model result reported in the paper.
